# Supplementary material for: Basal IFN-λ2/3 expression mediates tight junction formation in human epithelial cells
Source: EMBO J. 2025 Sep 1;44(20):5785–815. doi: 10.1038/s44318-025-00539-5 (PMC12528397; doi:10.1038/s44318-025-00539-5)
Supplement: Supplementary file 1 — Appendix [file 44318_2025_539_MOESM1_ESM.pdf]

Appendix for:

Basal IFN-λ2/3 expression mediates tight junction formation in human epithelial cells

Yagmur Keser[1#], Camila Metz-Zumaran[1,2,#], Zina M. Uckeley[1], Dorothee Reuss[2,3], Patricio Doldan[2], James M. Ramsden[1], Megan L. Stanifer[1*], Steeve Boulant[1,2*]

[1]Department of Molecular Genetics and Microbiology, College of Medicine, University of Florida, Gainesville, FL, USA

[2]Department of Infectious Disease, Virology, University Hospital Heidelberg, Heidelberg, Germany

[3]Current address: Department of Infectious Diseases, Imperial College London, London UK

[#]equal contribution

*Co-corresponding authors

Megan L. Stanifer, University of Florida, 1200 Newell Dr. Gainesville, FL 32601 m.stanifer@ufl.edu

Steeve Boulant, University of Florida, 1200 Newell Dr. Gainesville, FL 32601, s.boulant@ufl.edu

Table of Contents

Appendix Figure S1. Validation of IFNλ2/3 KO with Sanger sequencing. 3

Appendix Figure S2. H151 and IN-2 efficiently block cGAS/STING signaling. 4

Appendix Figure S3. Cytotoxicity analysis following inhibitor treatments. 5

Appendix Figure S4. Expression of housekeeping genes and ISGs across cell lines based on RNA-seq data. 6

Appendix Figure S5. Cell proliferation, cytotoxicity, apoptotic gene expression, and gene set enrichment analysis in WT, IFNLR1 KO, and IFNλ2/3 KO cells 8

Appendix Table S1: Compounds and concentrations for human organoid basal and differentiation media. 9

Appendix Table S2: List of primer sequences used in qPCR. 10

IFNλ2 gDNA sequence (242bp-421bp):

```
WT           5'- atgactggggactgcacgccagtgctggtgctgatggccgcagtgctgaccgtgactgga -3'
IFNλ2/3 KO   5'- atgactggggactgcacgccag-----------------gcagtgctgaccgtgactgga -3'

WT           5'- gcagttcctgtcgccaggctccacggggctctcccggatgcaaggggctgccacatagcc -3'
IFNλ2/3 KO   5'- gcagttcctgtcgccaggctccacggggctctcccggatgcaaggggctgccacatagcc -3'

WT           5'- cagttcaagtccctgtctccacaggagctgcaggcctttaagagggccaaagatgcctta -3'
IFNλ2/3 KO   5'- cagttcaagtccctgtctccacaggagctgcaggcctttaa -3'
```

IFNλ2 protein sequence (80aa-140aa):

```
WT           M  T  G  D  C  T  P  V  L  V  L  M  A  A  V  L  T  V  T  G
IFNλ2/3 KO   M  T  G  D  C  T  P  G  S  A  D  R  D  W  S  S  S  C  R  Q

WT           A  V  P  V  A  R  L  H  G  A  L  P  D  A  R  G  C  H  I  A
IFNλ2/3 KO   A  P  R  G  S  P  G  C  K  G  L  P  H  S  P  V  Q  V  P  V

WT           Q  F  K  S  L  S  P  Q  E  L  Q  A  F  K  R  A  K  D  A  L
IFNλ2/3 KO   S  T  G  A  A  G  L  *
```

IFNλ3 gDNA sequence (1bp-180bp):

```
WT           5'- atgaccggggactgcatgccagtgctggtgctgatggccgcagtgctgaccgtgactgga -3'
IFNλ2/3 KO   5'- atgaccggggactgcatgccagtgctggtgctgatggccgcagtgctgaccgtgactgga -3'

WT           5'- gcagttcctgtcgccaggctccgcggggctctcccggatgcaaggggctgccacatagcc -3'
IFNλ2/3 KO   5'- gcagttc-----gccaggctccgcggggctctcccggatgcaaggggctgccacatagcc -3'

WT           5'- cagttcaagtccctgtctccacaggagctgcaggcctttaagagggccaaagatgcctta -3'
IFNλ2/3 KO   5'- cagttcaagtccctgtctccacaggagctgcaggcctttaa -3'
```

IFNλ3 protein sequence (1aa-60aa):

```
WT           M  T  G  D  C  M  P  V  L  V  L  M  A  A  V  L  T  V  T  G
IFNλ2/3 KO   M  T  G  D  C  M  P  V  L  V  L  M  A  A  V  L  T  V  T  G

WT           A  V  P  V  A  R  L  R  G  A  L  P  D  A  R  G  C  H  I  A
IFNλ2/3 KO   A  V  R  Q  A  P  R  G  S  P  G  C  K  G  L  P  H  S  P  V

WT           Q  F  K  S  L  S  P  Q  E  L  Q  A  F  K  R  A  K  D  A  L
IFNλ2/3 KO   Q  V  P  V  S  T  G  A  A  G  L  *
```

Appendix Figure S1. Validation of IFNλ2/3 KO with Sanger sequencing. Genomic DNA from T84 WT and IFNλ2/3 KO cells were isolated. PCR was performed for the amplification of IFNλ2 and IFNλ3 loci. PCR product was purified from agarose gel electrophoresis and sequenced by Sanger sequencing. The Basic Local Alignment Search Tool (BLAST) was used to align the resulting sequencing.

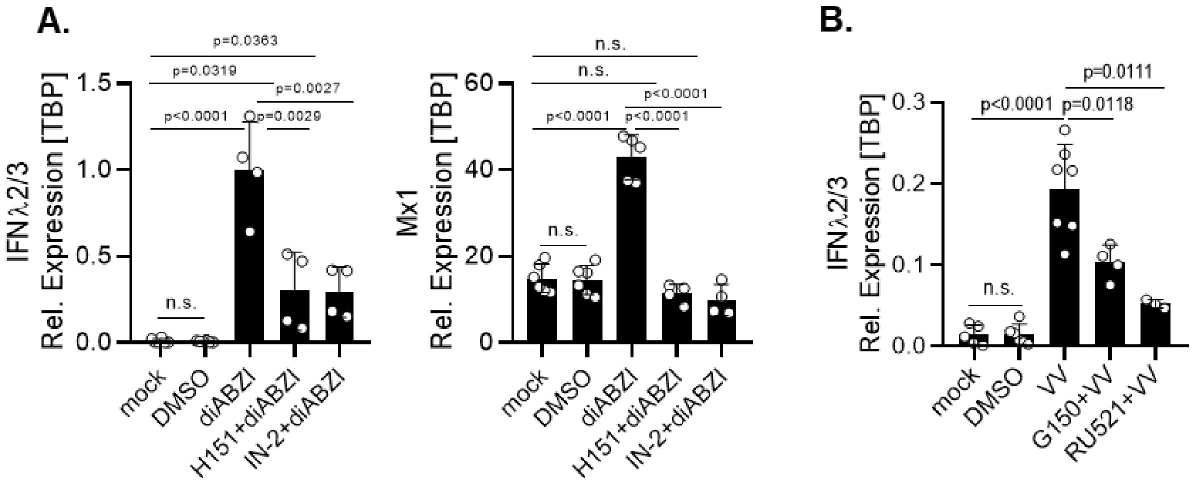

Appendix Figure S2. H151 and IN-2 efficiently block cGAS/STING signaling. (A) T84 WT cells were treated with either culture media (mock) or DMSO as a control or treated with 10 µM of the STING-agonist diABZI alone or in combination with the STING-inhibitors H151 (20 µM) or IN-2 (2 µM). Induction of IFNλ2/3 and Mx1 expression by diABZI was monitored using q-RT-PCR. **(B)** T84 WT cells were treated with either culture media (mock) or DMSO (10 µM) as a control or infected with Vaccinia virus (VV) alone or in combination with the cGAS-inhibitors G150 or RU521 at 10 µM. Induction of IFNλ2/3 expression by Vaccinia virus was monitored using q-RT-PCR. The relative expression of IFNλ2/3 and Mx1 was normalized to TBP. n ≥ 3 biological replicates. Statistical analysis was performed using ordinary two-way ANOVA. n.s. indicates non-significant results ($p > 0.05$). Exact *p*-values are shown on the plots when significant; otherwise, results are not significant. Error bars represent standard deviation with the mean as the center.

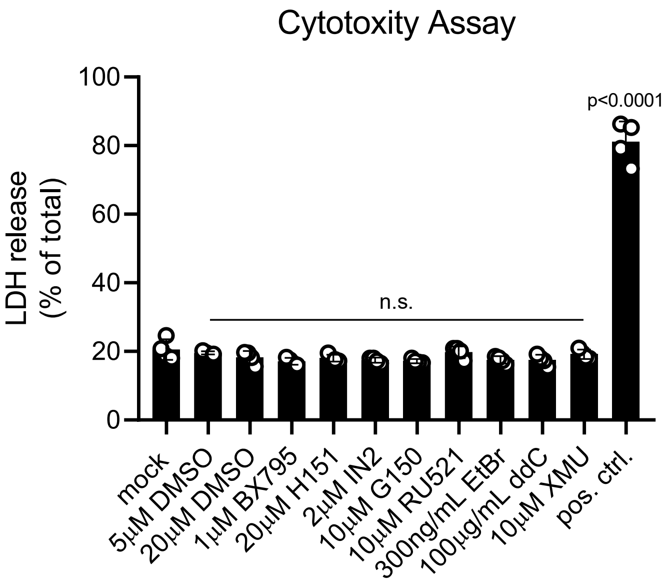

Appendix Figure S3. Cytotoxicity analysis following inhibitor treatments. Cytotoxicity was measured in cells treated with the indicated inhibitors. LDH release into the culture supernatant was quantified and normalized to total intracellular LDH to calculate percent cytotoxicity. Treatment with 50 µM PPMP served as the positive control (pos. ctrl.). $n \geq 3$ biological replicates. Statistical analysis was performed using ordinary one-way ANOVA. n.s. indicates non-significant results ($p > 0.05$). Exact p-values are shown on the plots when significant; otherwise, results are not significant. Error bars represent standard deviation with the mean as the center.

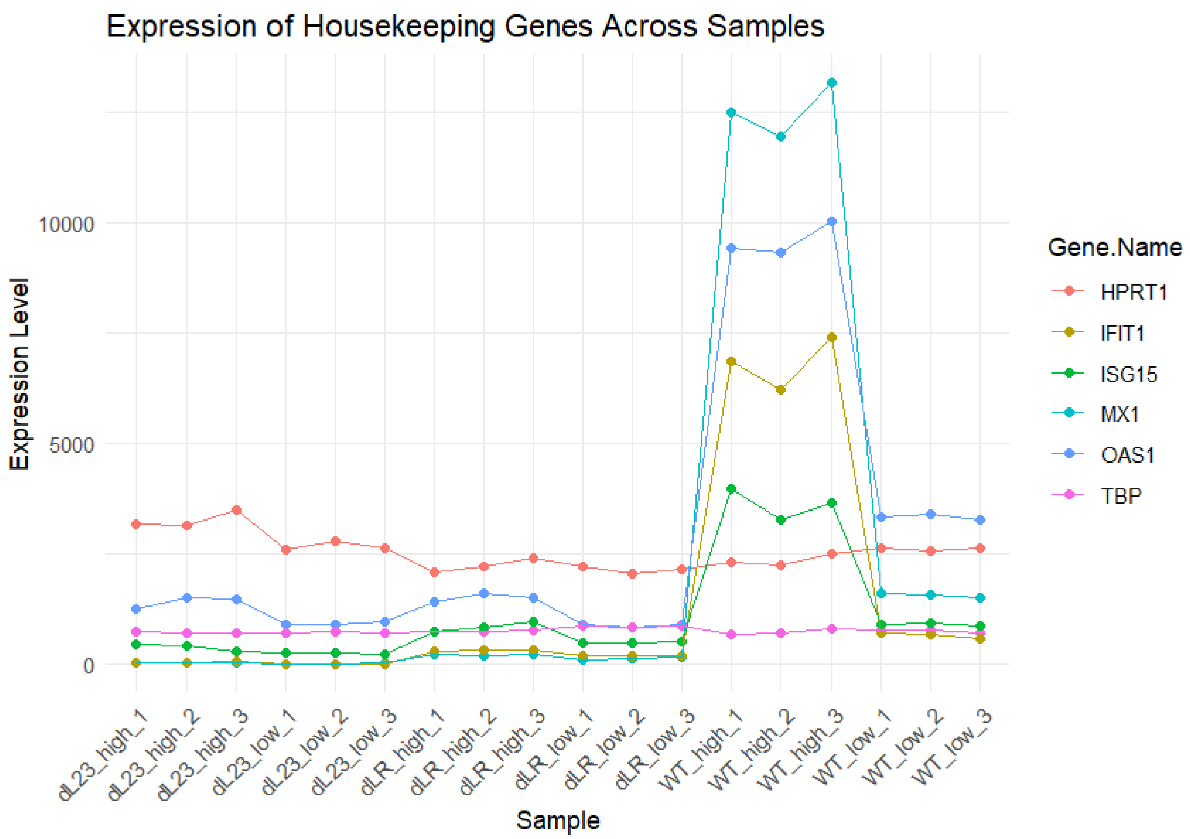

Appendix Figure S4. Expression of housekeeping genes and ISGs across cell lines based on RNA-seq data. Expression levels of selected housekeeping genes (HPRT1, TBP) and ISGs (IFIT1, ISG15, MX1, OAS1) were analyzed across indicated samples using RNA sequencing. Each point represents an individual biological replicate. Expression levels are presented as normalized raw counts.

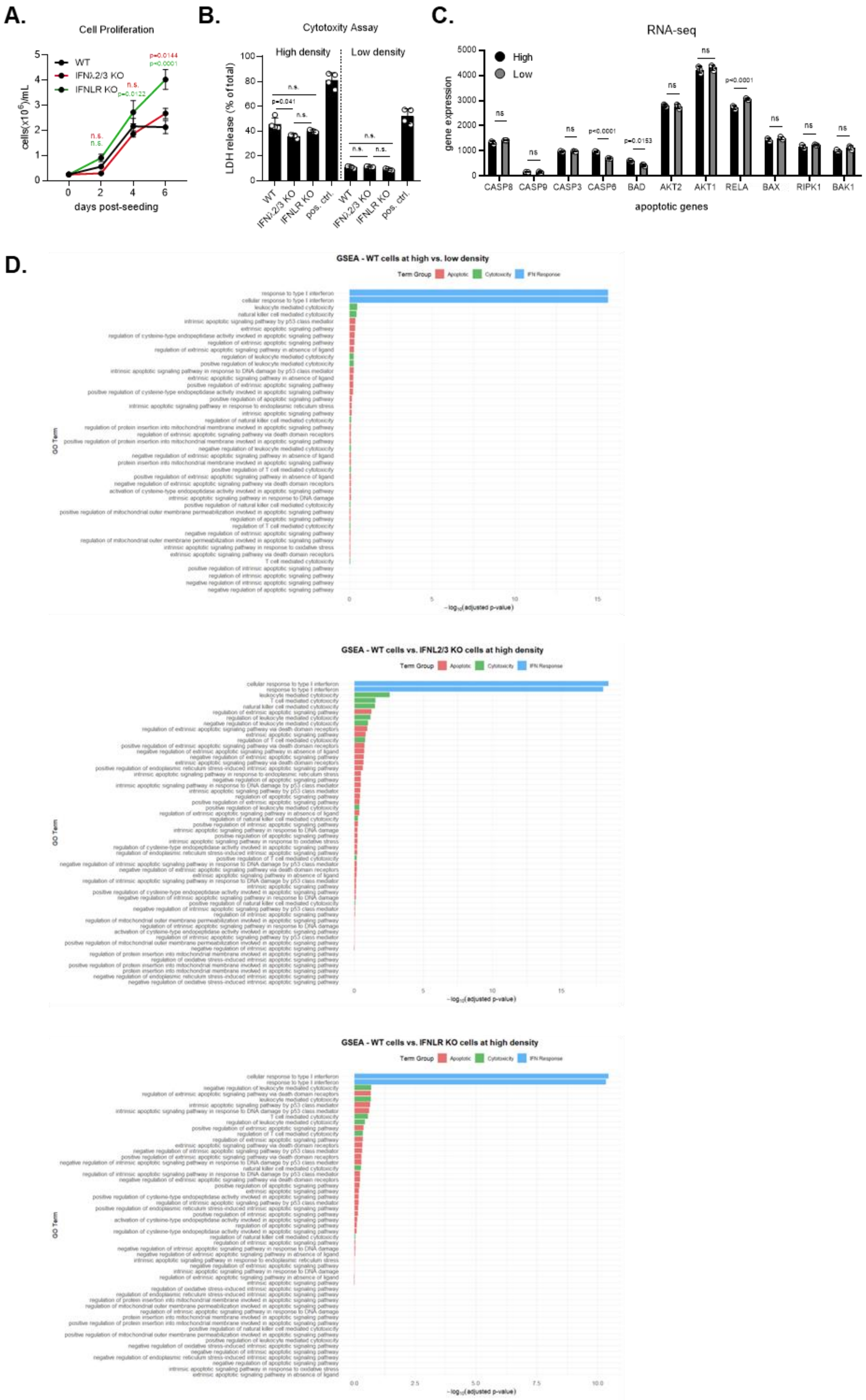
A.
Cell Proliferation
WT
IFNλ2/3 KO
IFNLR KO
p=0.0144
p=0.0001
n.s.
p=0.0122
n.s.
n.s.
cells(x10^6)/mL
days post-seeding
B.
Cytotoxity Assay
High density
Low density
p=0.041
n.s.
LDH release (% of total)
WT
IFNλ2/3 KO
IFNLR KO
pos. ctrl.
C.
RNA-seq
High
Low
gene expression
ns
p=0.0001
p=0.0153
CASP8
CASP9
CASP3
CASP6
BAD
AKT2
AKT1
RELA
BAX
RIPK1
BAK1
apoptotic genes
D.
GSEA - WT cells at high vs. low density
Term Group
Apoptotic
Cytotoxicity
IFN Response
GO Term
-log10(adjusted p-value)
GSEA - WT cells vs. IFNL2/3 KO cells at high density
GSEA - WT cells vs. IFNLR KO cells at high density

Appendix Figure S5. Cell proliferation, cytotoxicity, apoptotic gene expression, and gene set enrichment analysis in WT, IFNLR1 KO, and IFNλ2/3 KO cells. (A) T84 WT, IFNλ2/3 and IFNLR KO cells were seeded into a 12-well plate at a density of 3.6 x 10^4 cells per well. Every second day, the cells were detached, and the live cell count was determined. **(B)** Cytotoxicity was assessed by measuring LDH release in high- and low-density cultures of WT, IFNλ2/3 KO and IFNLR KO cells. Positive control (pos. ctrl.) indicates cells treated with 50 μM PPMP to induce cytotoxicity. **(C)** Expression of selected apoptotic genes (CASP8, CASP9, CASP3, BAD, AKT2, AKT1, RELA, BAX, RIPK1, BAK1) was compared between high- and low-density WT cells using RNA sequencing. Expression levels are presented as normalized raw counts. **(B, C)** n ≥ 3 biological replicates. Statistical analysis was performed using ordinary two-way ANOVA. n.s. indicates non-significant results ($p > 0.05$). Exact *p*-values are shown on the plots when significant; otherwise, results are not significant. Error bars represent standard deviation with the mean as the center. **(D)** Gene Ontology (GO) terms categorized into IFN response (blue), cytotoxicity (green), and apoptotic pathways (red) were analyzed based on RNA-seq data. Top panel: Comparison of WT cells at high vs. low density. Middle panel: Comparison of WT vs. IFNλ2/3 KO cells at high density. Bottom panel: Comparison of WT vs. IFNLR KO cells at high density.

Basal media		Differentiation media	
**Compound**	**Final concentration**	**Compound**	**Final concentration**
Advanced DMEM/F12 (Gibco #12634010) + 2 mM GlutaMAX (Gibco #35050061) + 10 mM HEPES (Sigma-Aldrich #H7523) + 100 U/mL penicillin and 100 µg/mL streptomycin		Advanced DMEM/F12 + 1x GlutaMAX + 10 mM HEPES +100 U/mL penicillin and 100 µg/mL streptomycin	
L-WRN cell conditioned supernatant (WNT, R-Spondin, Noggin)	62.5% (v/v)	R-Spondin cell conditioned supernatant (WNT, R-Spondin, Noggin)	10.5% (v/v)
B-27 Supplement (Thermo Fisher #17504001)	1x	B-27 Supplement	1x
EGF (recombinant mouse) (Gibco #PMG8041)	50 ng/mL	EGF (recombinant mouse)	50 ng/mL
A83-01 (Millipore Sigma #SML0788)	500 nM	A83-01	500 nM
IGF-1 (recombinant human) (Thermo Fisher #590908)	100 ng/mL	IGF-1 (recombinant human)	100 ng/mL
FGF-basic (recombinant human) (Peprotech # 100-18B)	50 ng/mL	FGF-basic (recombinant human)	75 ng/mL
Noggin (recombinant mouse) (Peptrotech #250-38-100UG)	25 ng/mL	Noggin (recombinant mouse)	50 ng/mL
Gastrin (Sigma-Aldrich # G9145)	10 nM	Gastrin	10 nM
N-acetyl-cysteine (Sigma-Aldrich #A9165)	1 mM		

Appendix Table S1: Compounds and concentrations for human organoid basal and differentiation media.

Target Gene	Forward sequence (5'→3')	Reverse sequence (5'→3')
Human 18S	AGAGGGACAAGTGGCGTTC	CGCTGAGCCAGTCAGTGT
Human Claudin-2	CTGCTTTTCCTGCTCATCCC	AGAGCTCCTTGTGGCAAGAGG
Human IFIT1	AAAAGCCCACATTTGAGGTG	GAAATTCCTGAAACCGACCA
Human IFNβ	GCCGCATTGACCATCTAT	GTCTCATTCCAGCCAGTG
Human IFNλ2/3	GCCACATAGCCCAGTTCAAG	TGGGAGAGGATATGGTGCAG
Human IFNAR1	ATCGGTGCTCCAAAACAGTC	GTGCTCTGGCTTTCACACAA
Human IFNLR	ATCCTCAGTTAACCTACACC	CAGATACTCCACCACAAAAC
Human IL-1β	CCACAGACCTTCCAGGAGAATG	GTGCAGTTCAGTGATCGTACAGG
Human IL-6	GCACTGGCAGAAAACAACCT	TCAAACTCCAAAAGACCAGTGA
Human IL-8	GAGAGTGATTGAGAGTGGACCAC	CACAACCCTCTGCACCCAGTTT
Human IL-18	GATAGCCAGCCTAGAGGTATGG	CCTTGATGTTATCAGGAGGATTCA
Human ISG15	CCTCTGAGCATCCTGGT	AGGCCGTACTCCCCCAG
Human nd2	CACCCAAGAACAGGGTTTGT	TGGCCATGGGTATGTTGTTAA
Human Mx1	GAGCTGTTCTCCTGCACCTC	CTCCCACTCCCTGAAATCTG
Human OAS1	TGCGCTCAGCTTCGTACTGA	GGTGGAGAACTCGCCCTCTT
Human TBP	CCACTCACAGACTCTCACAAC	CTGCGGTACAATCCCAGAACT
Human Viperin	GAGAGCCATTTCTTCAAGACC	CTATAATCCCTACACCACCTCC

Appendix Table S2: List of primer sequences used in qPCR.
