# Supplementary figures and images for: Basal IFN-λ2/3 expression mediates tight junction formation in human epithelial cells
Source: EMBO J. 2025 Sep 1;44(20):5785–815. doi: 10.1038/s44318-025-00539-5 (PMC12528397; doi:10.1038/s44318-025-00539-5)

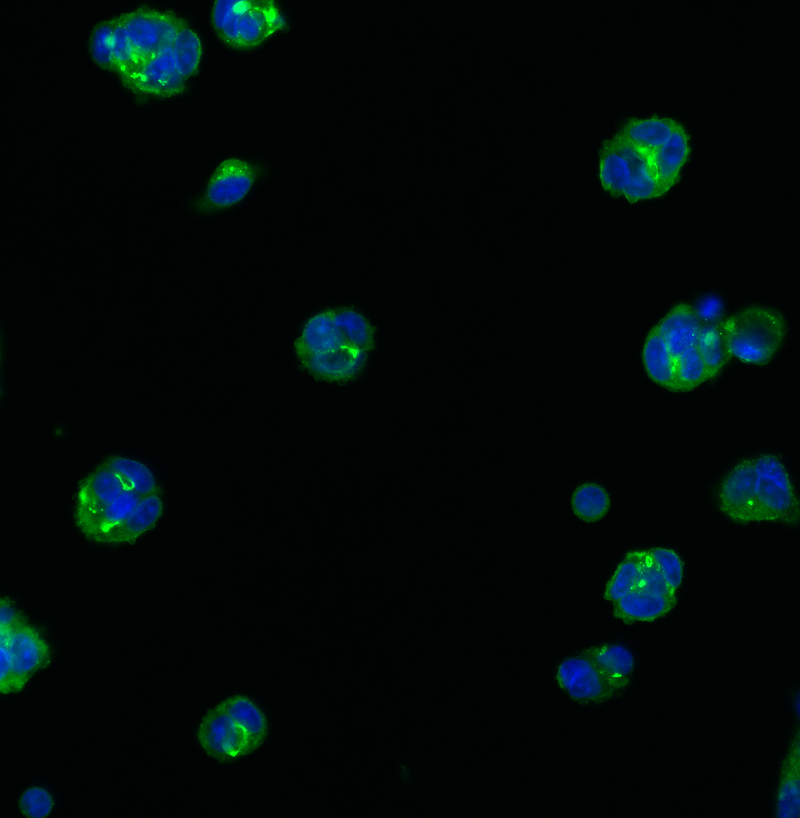

Supplement: Supplementary file 3 — Source data Fig. 1 [file 44318_2025_539_MOESM3_ESM.zip › Figure 1/Figure 1C/Figure 1C_Day1.tiff]

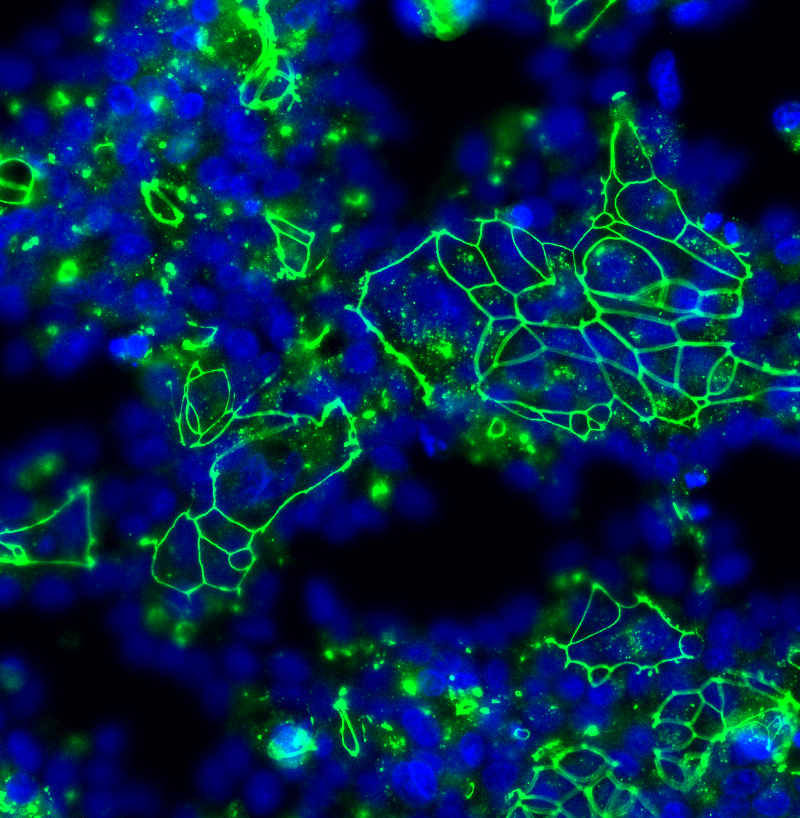

Supplement: Supplementary file 3 — Source data Fig. 1 [file 44318_2025_539_MOESM3_ESM.zip › Figure 1/Figure 1C/Figure 1C_Day4.tiff]

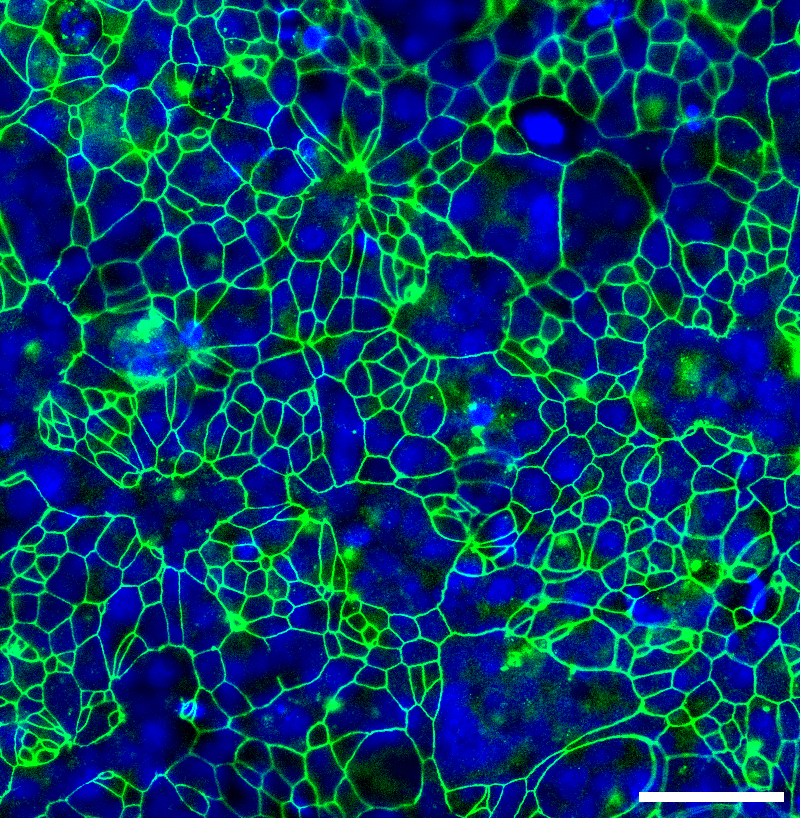

Supplement: Supplementary file 3 — Source data Fig. 1 [file 44318_2025_539_MOESM3_ESM.zip › Figure 1/Figure 1C/Figure 1C_Day8.tiff]

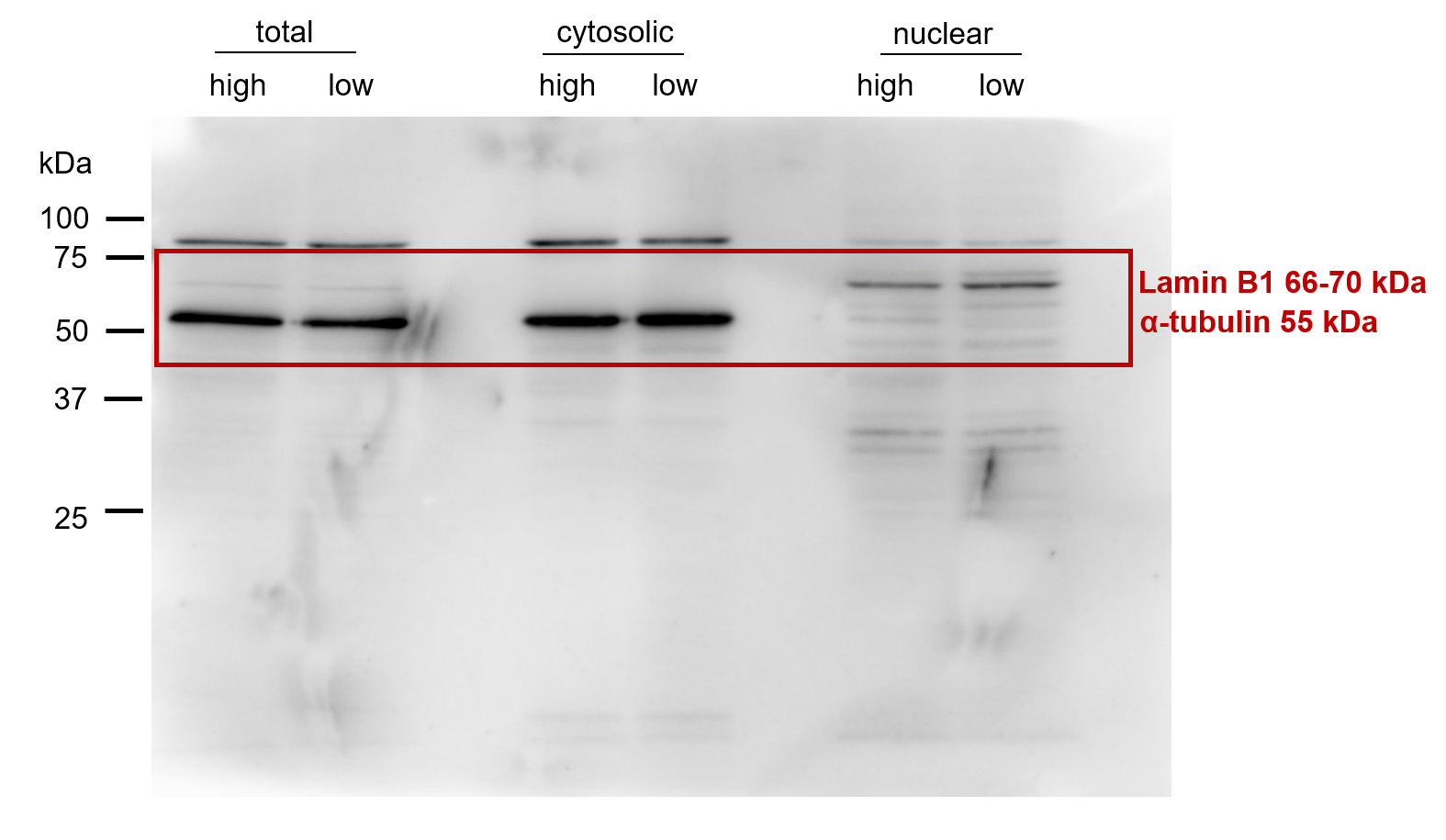

Supplement: Supplementary file 4 — Source data Fig. 2 [file 44318_2025_539_MOESM4_ESM.zip › Figure 2/Figure 2A/Western LaminB1 and Tubulin.tif]

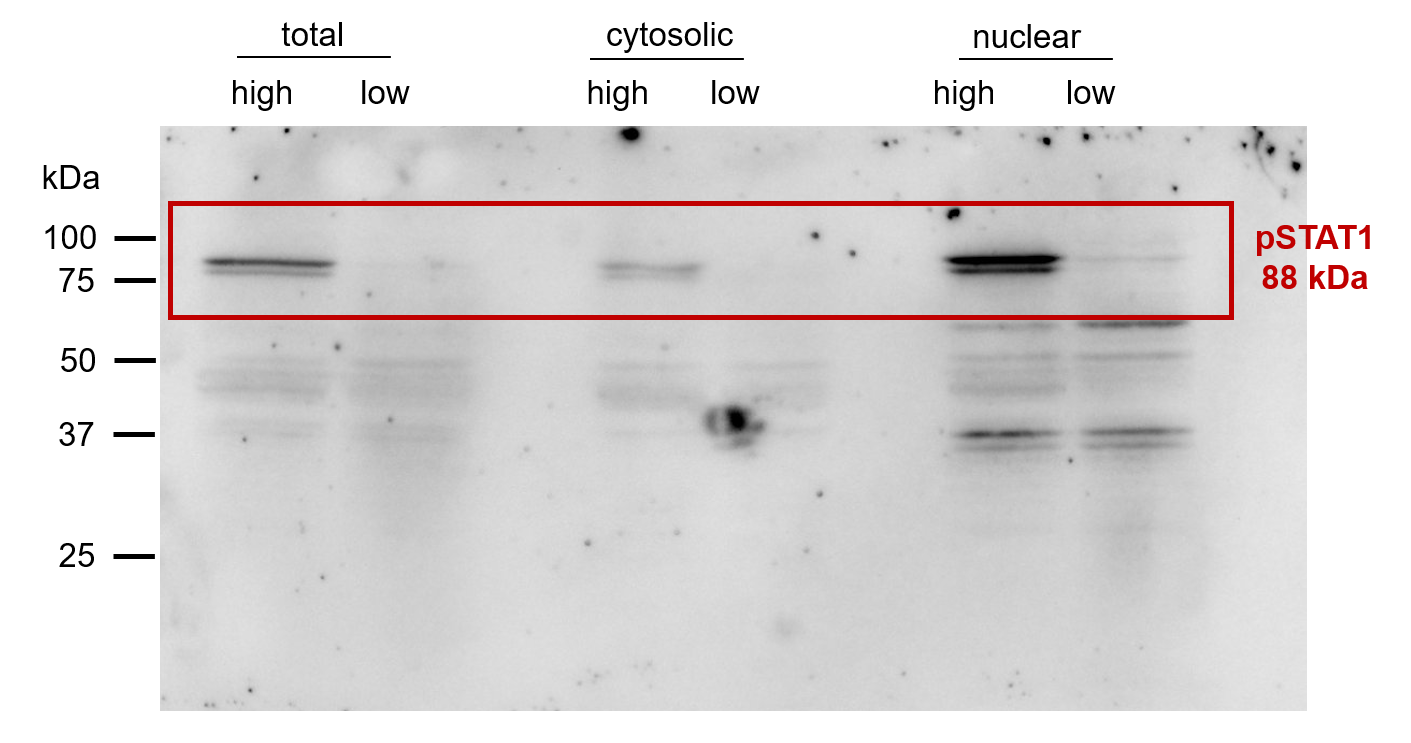

Supplement: Supplementary file 4 — Source data Fig. 2 [file 44318_2025_539_MOESM4_ESM.zip › Figure 2/Figure 2A/Western pSTAT1.tif]

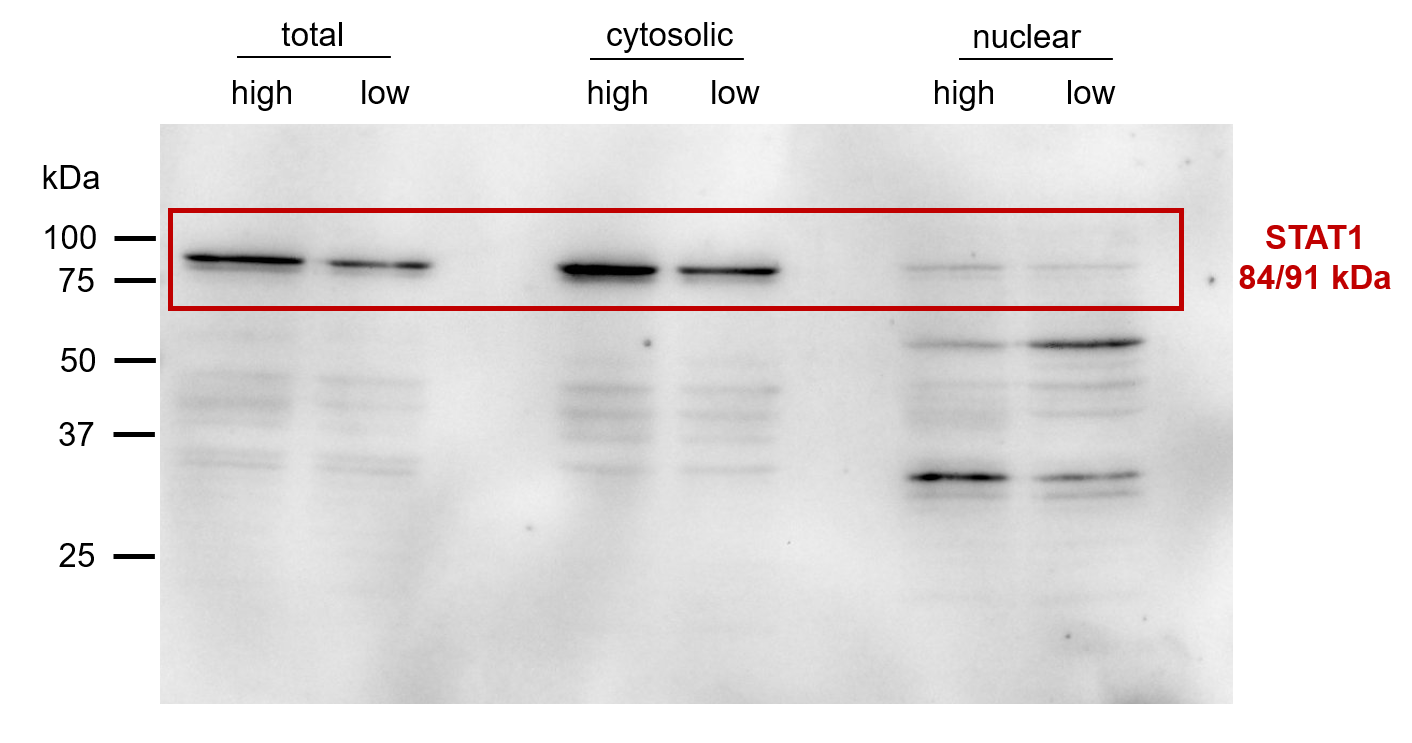

Supplement: Supplementary file 4 — Source data Fig. 2 [file 44318_2025_539_MOESM4_ESM.zip › Figure 2/Figure 2A/Western STAT1.tif]

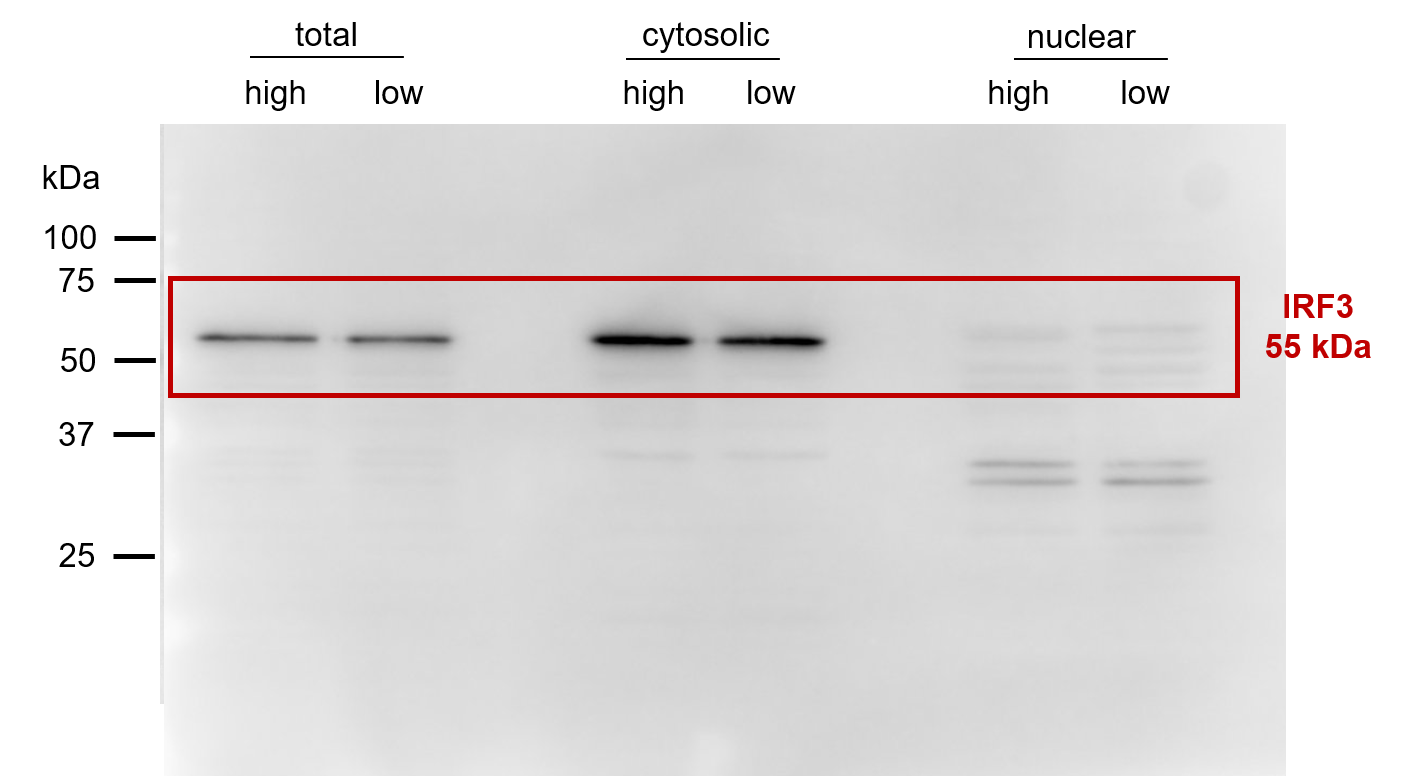

Supplement: Supplementary file 4 — Source data Fig. 2 [file 44318_2025_539_MOESM4_ESM.zip › Figure 2/Figure 2C/Western IRF3.tif]

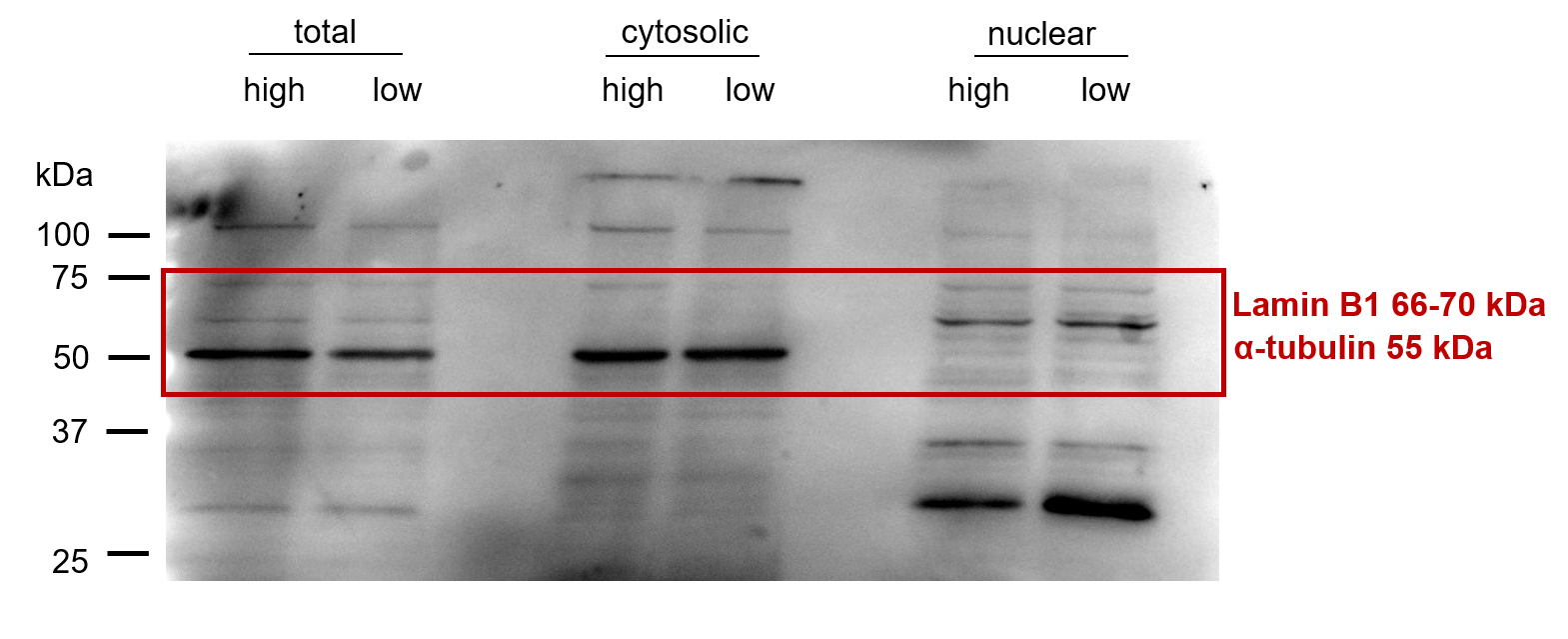

Supplement: Supplementary file 4 — Source data Fig. 2 [file 44318_2025_539_MOESM4_ESM.zip › Figure 2/Figure 2C/Western LaminB1 and Tubulin.tif]

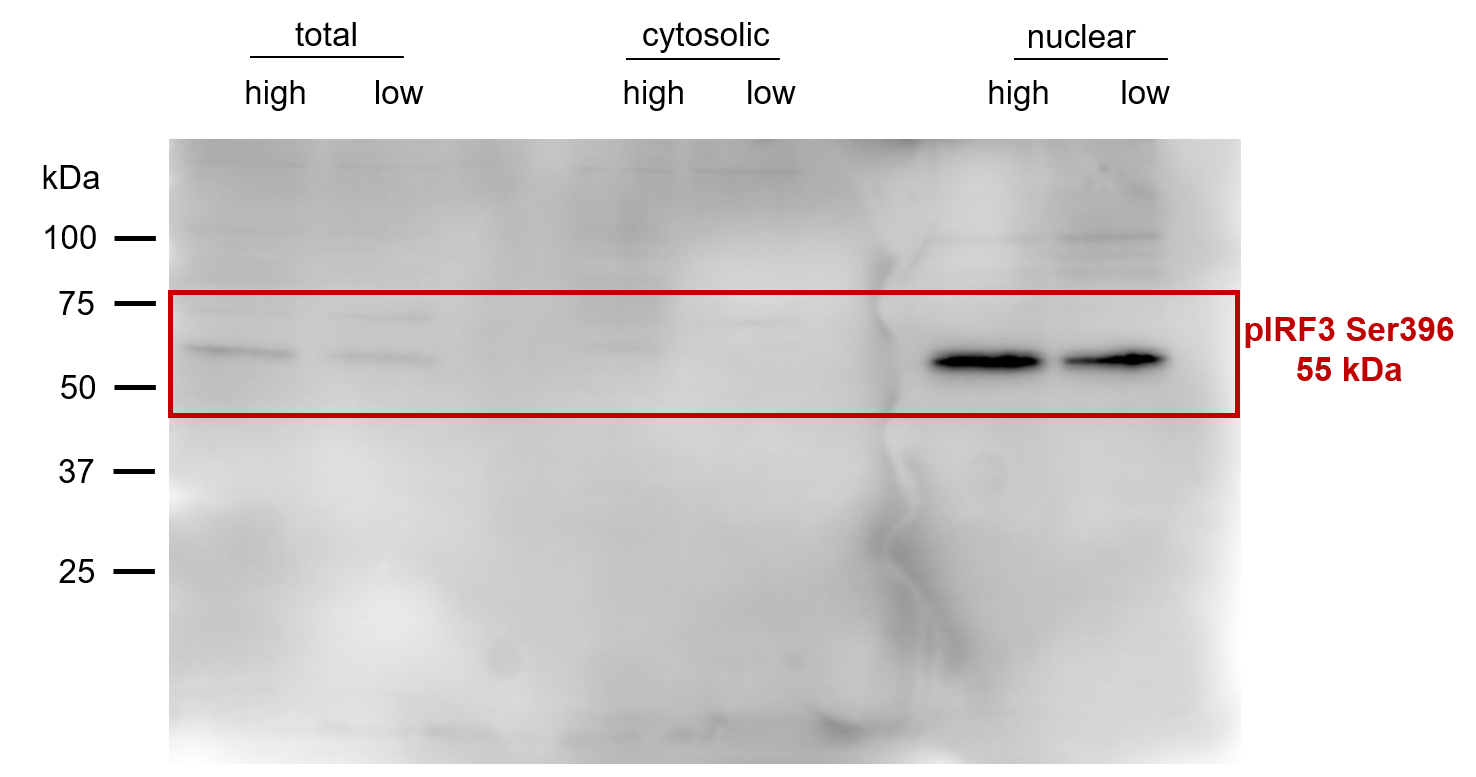

Supplement: Supplementary file 4 — Source data Fig. 2 [file 44318_2025_539_MOESM4_ESM.zip › Figure 2/Figure 2C/Western pIRF3.tif]

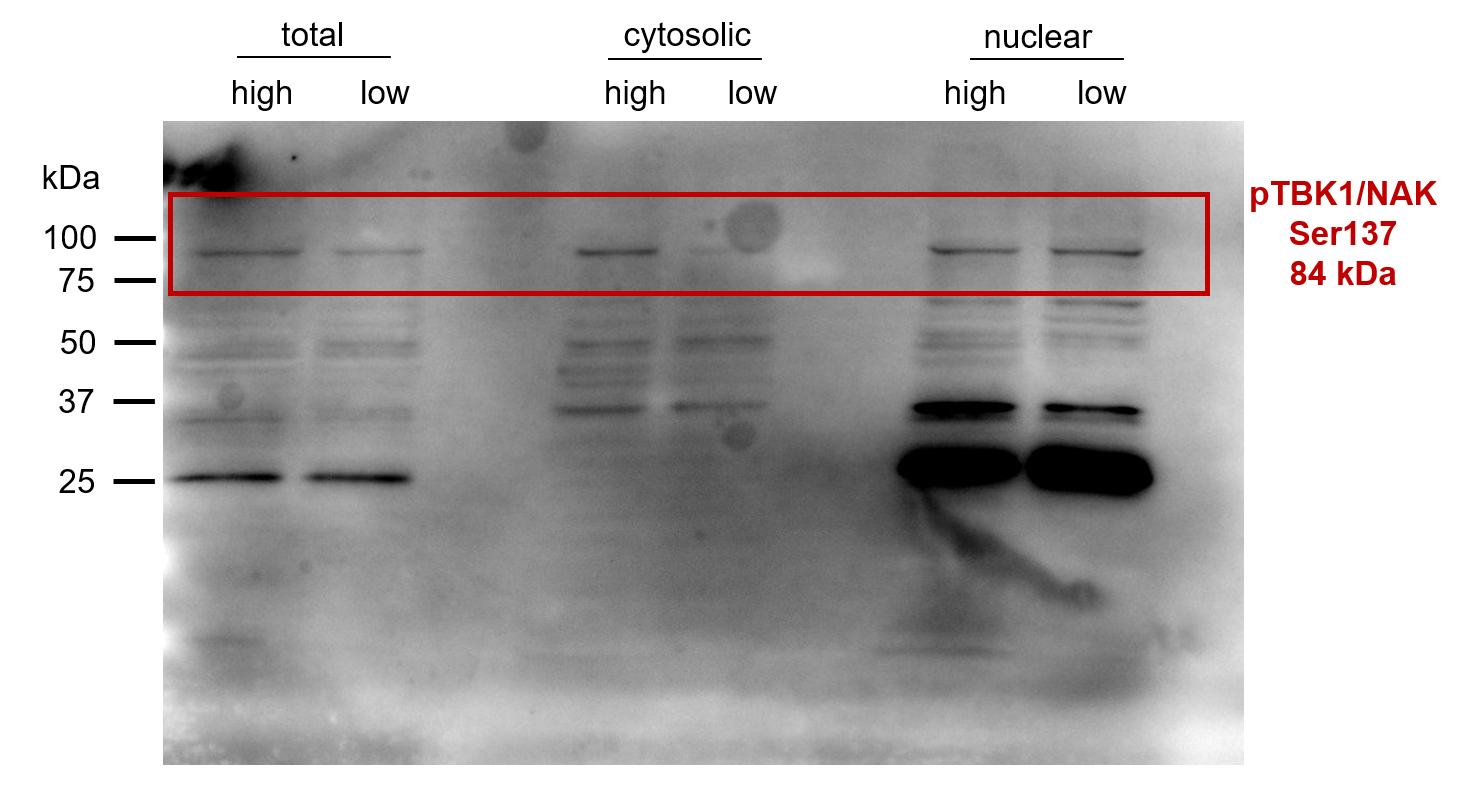

Supplement: Supplementary file 4 — Source data Fig. 2 [file 44318_2025_539_MOESM4_ESM.zip › Figure 2/Figure 2C/Western pTBK1 NAK.tif]

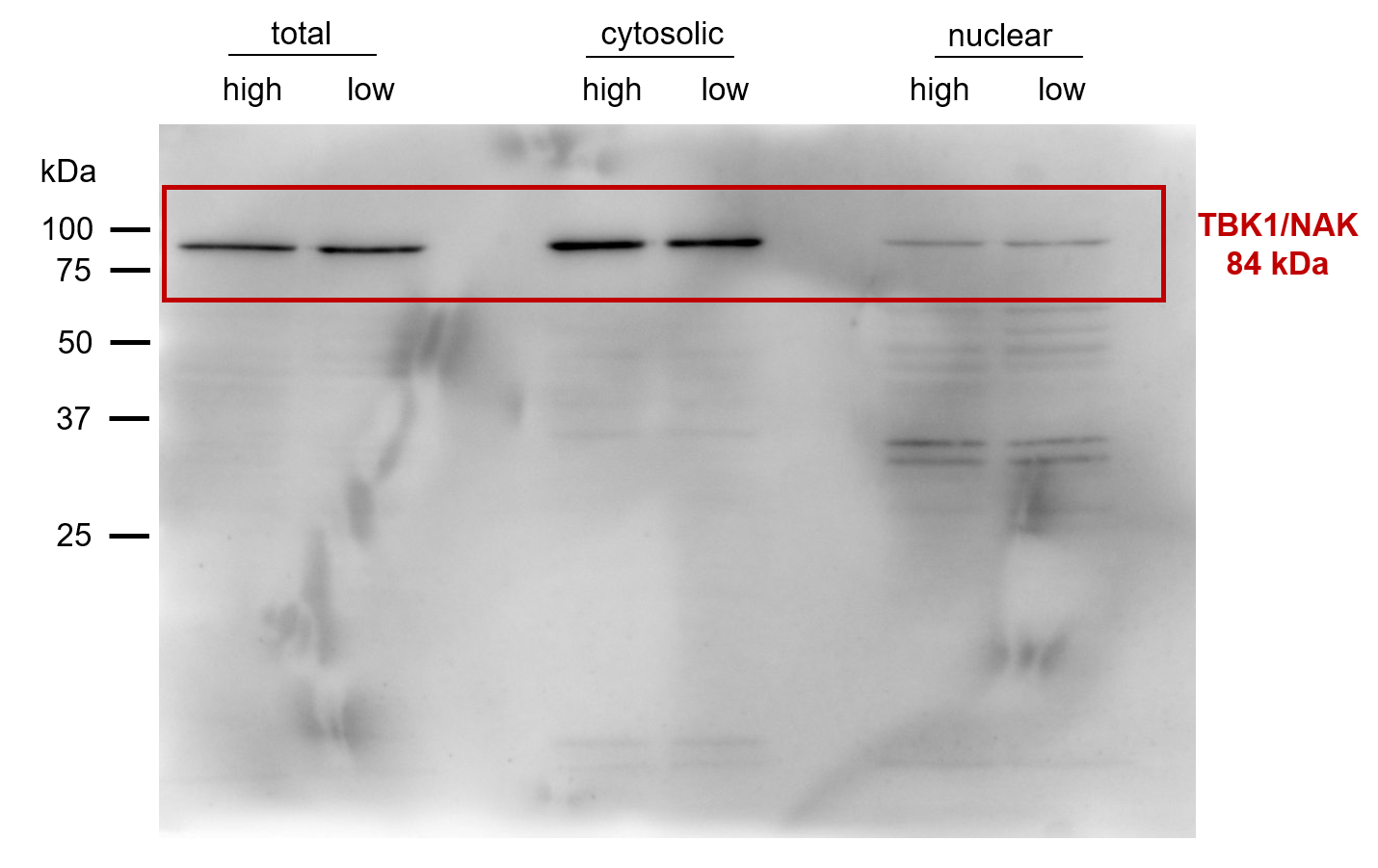

Supplement: Supplementary file 4 — Source data Fig. 2 [file 44318_2025_539_MOESM4_ESM.zip › Figure 2/Figure 2C/Western TBK1 NAK.tif]

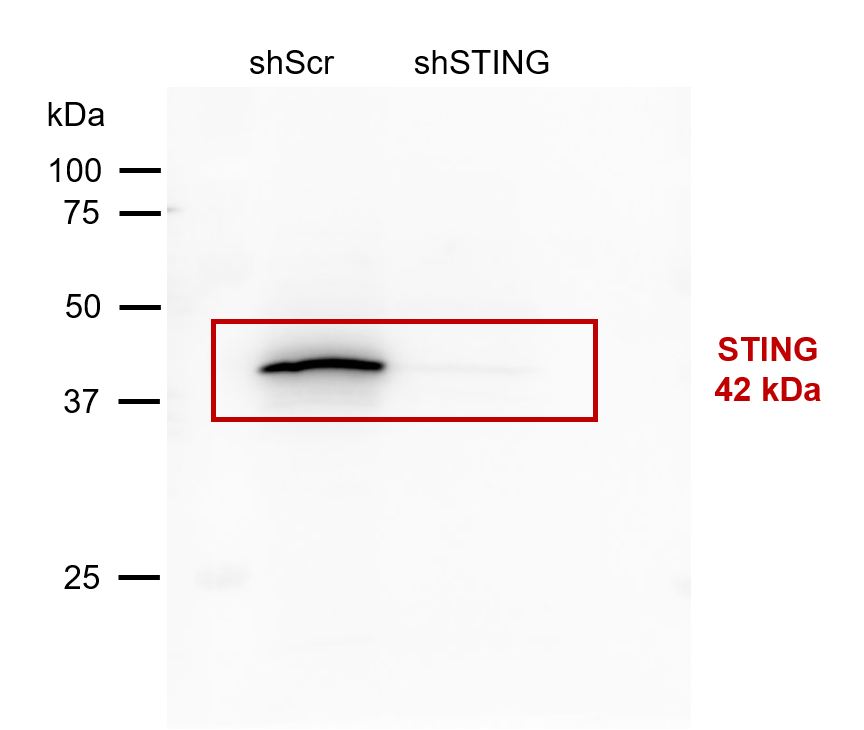

Supplement: Supplementary file 5 — Source data Fig. 3 [file 44318_2025_539_MOESM5_ESM.zip › Figure 3/Figure 3B/Western STING.tif]

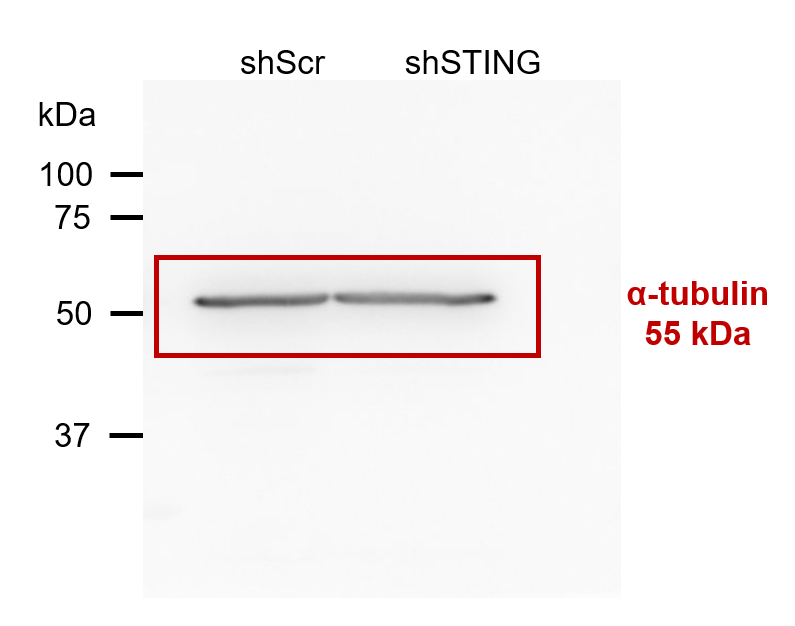

Supplement: Supplementary file 5 — Source data Fig. 3 [file 44318_2025_539_MOESM5_ESM.zip › Figure 3/Figure 3B/Western Tubulin.tif]

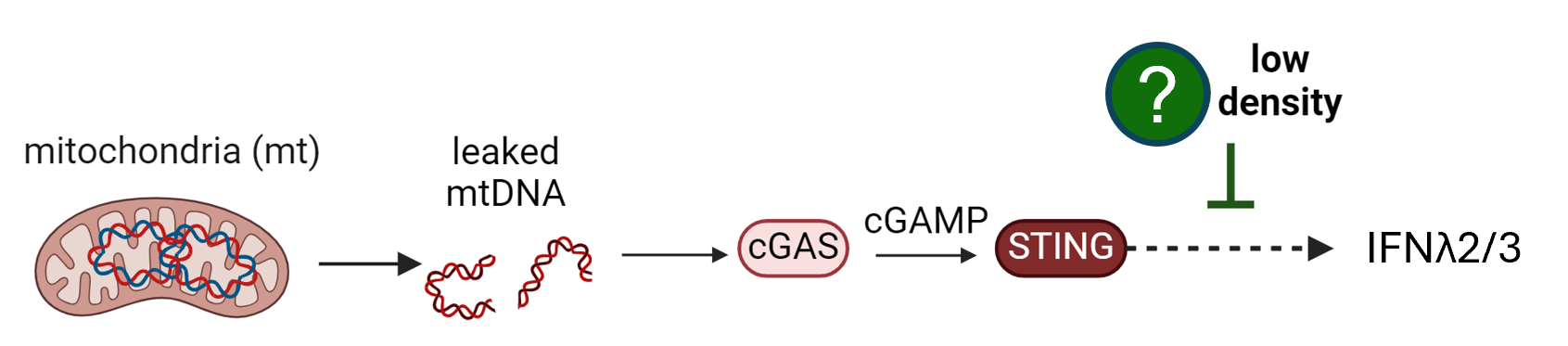

Supplement: Supplementary file 5 — Source data Fig. 3 [file 44318_2025_539_MOESM5_ESM.zip › Figure 3/Figure 3I/Figure 3I.tif]

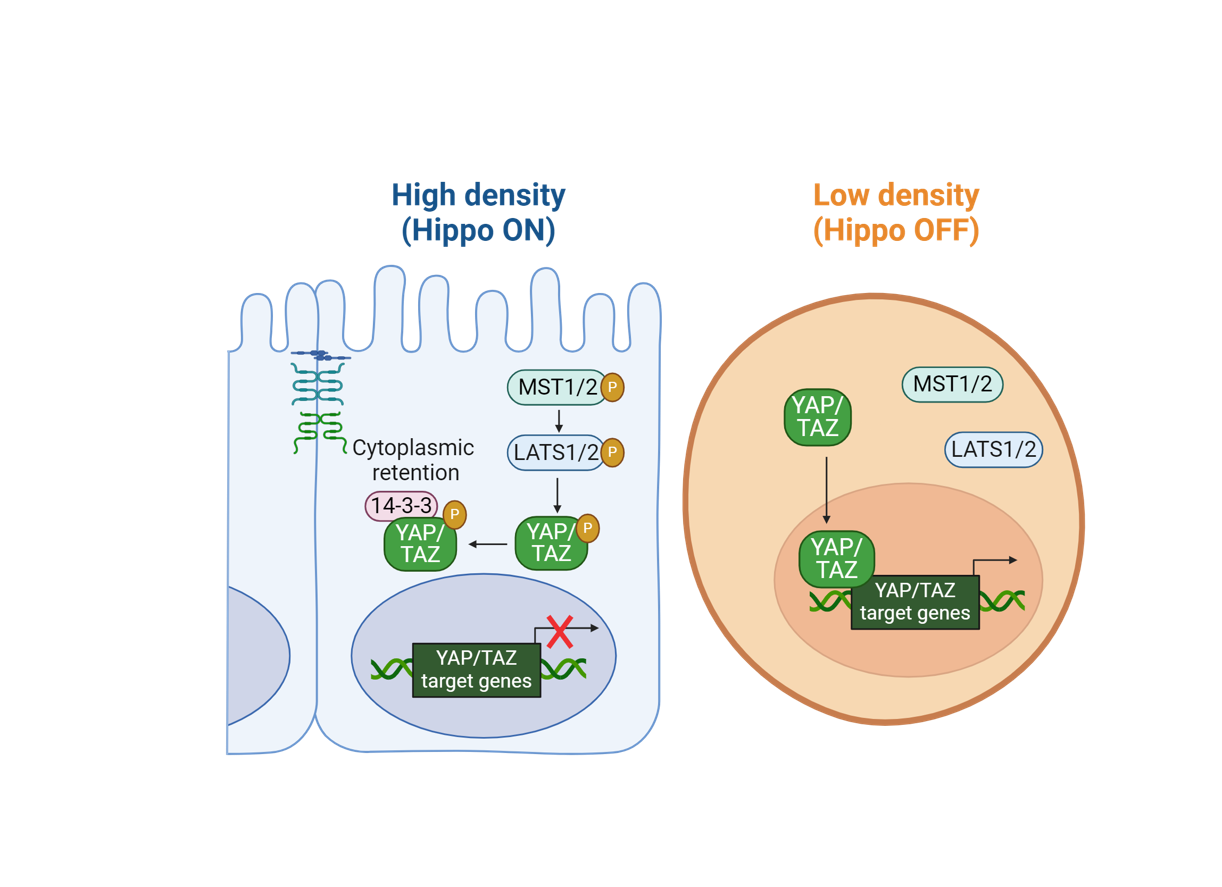

Supplement: Supplementary file 6 — Source data Fig. 4 [file 44318_2025_539_MOESM6_ESM.zip › Figure 4/Figure 4A/Figure 4A.tif]

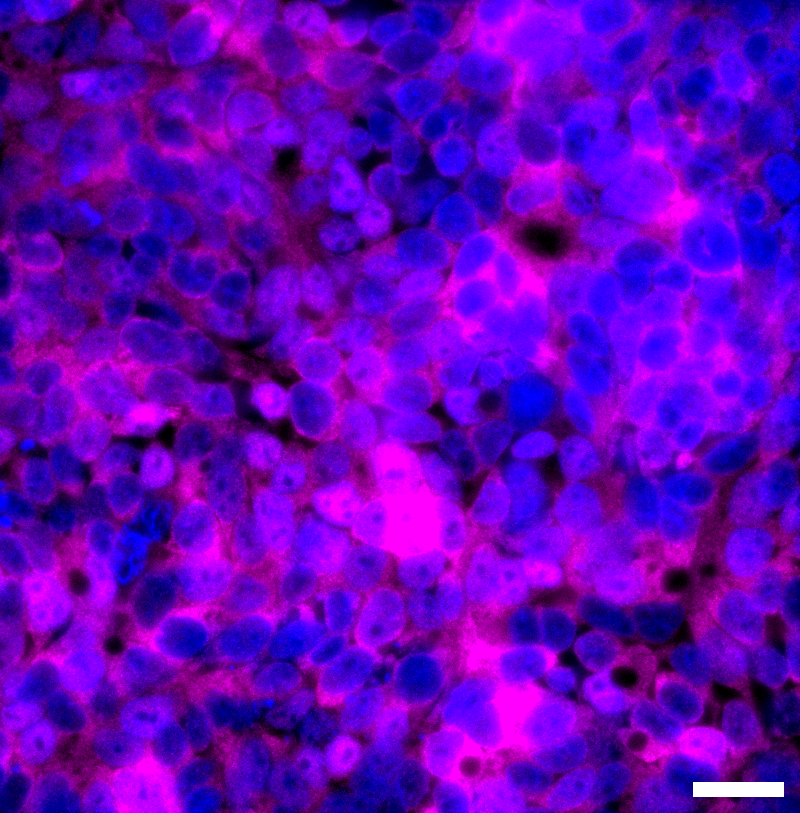

Supplement: Supplementary file 6 — Source data Fig. 4 [file 44318_2025_539_MOESM6_ESM.zip › Figure 4/Figure 4B/Figure 4B_high density.tif]

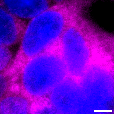

Supplement: Supplementary file 6 — Source data Fig. 4 [file 44318_2025_539_MOESM6_ESM.zip › Figure 4/Figure 4B/Figure 4B_high density_zoom.tif]

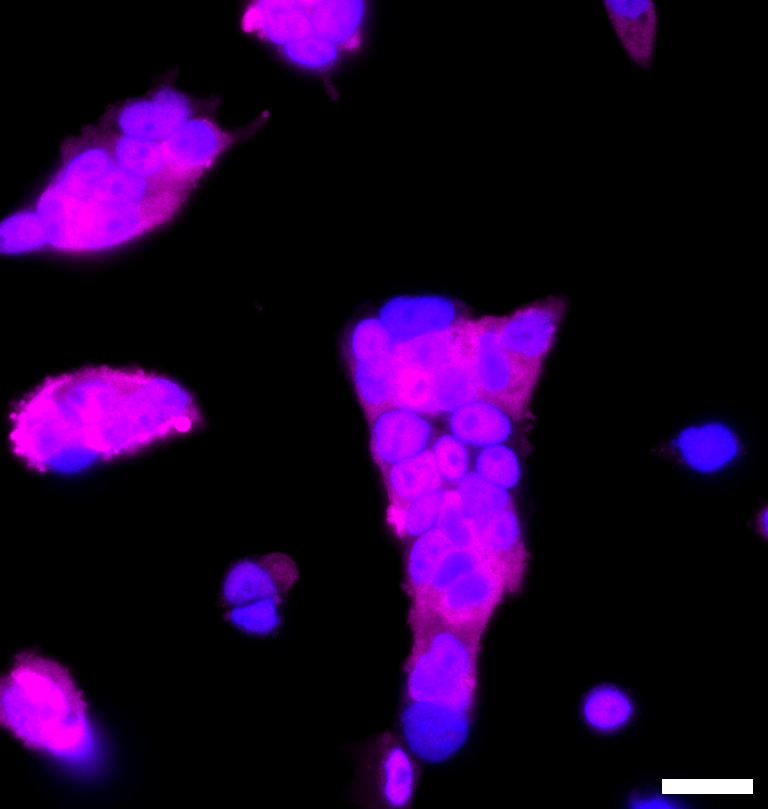

Supplement: Supplementary file 6 — Source data Fig. 4 [file 44318_2025_539_MOESM6_ESM.zip › Figure 4/Figure 4B/Figure 4B_low density.tif]

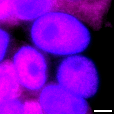

Supplement: Supplementary file 6 — Source data Fig. 4 [file 44318_2025_539_MOESM6_ESM.zip › Figure 4/Figure 4B/Figure 4B_low density_zoom.tif]

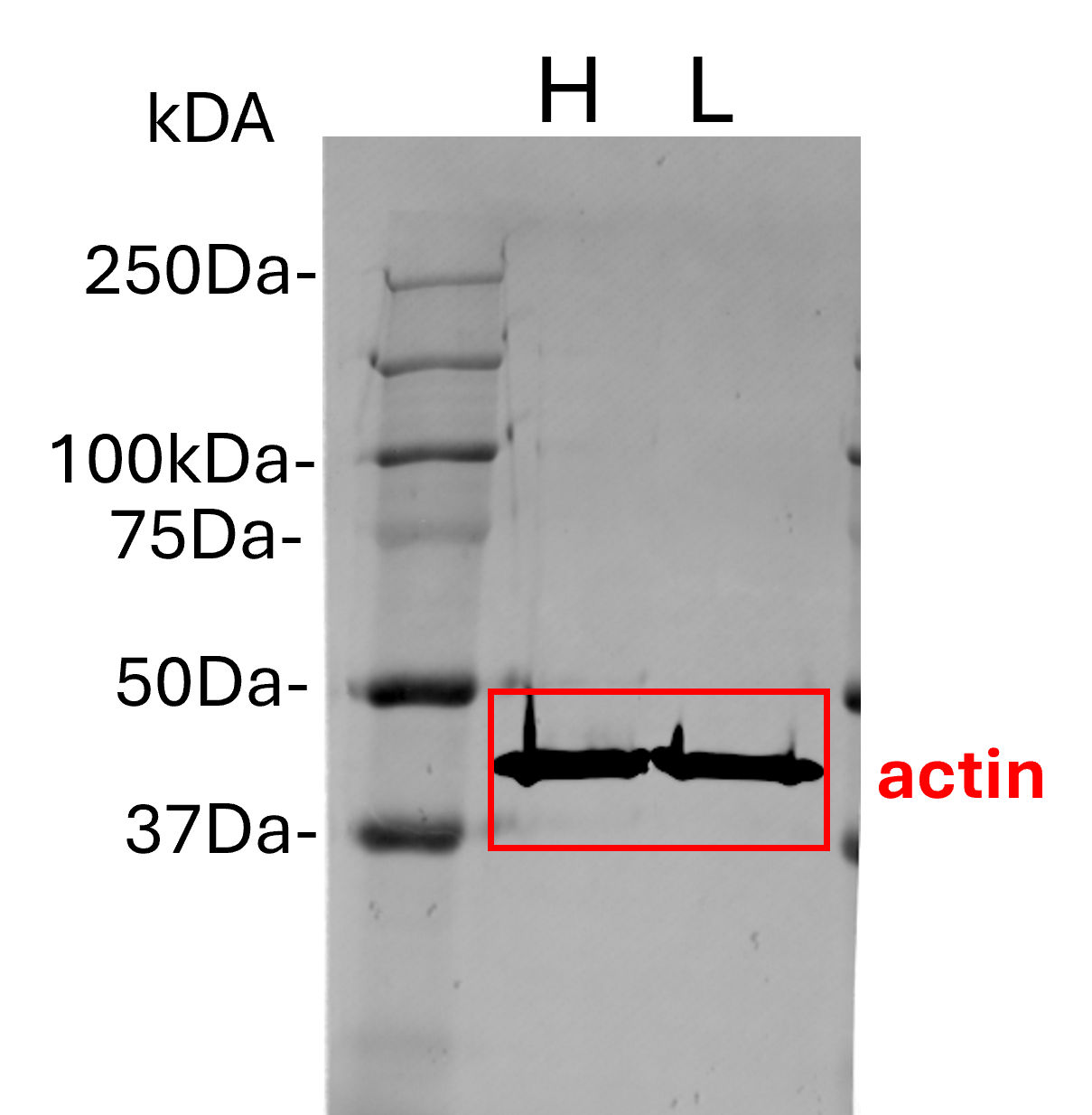

Supplement: Supplementary file 6 — Source data Fig. 4 [file 44318_2025_539_MOESM6_ESM.zip › Figure 4/FIgure 4C/Figure 4C_actin.tif]

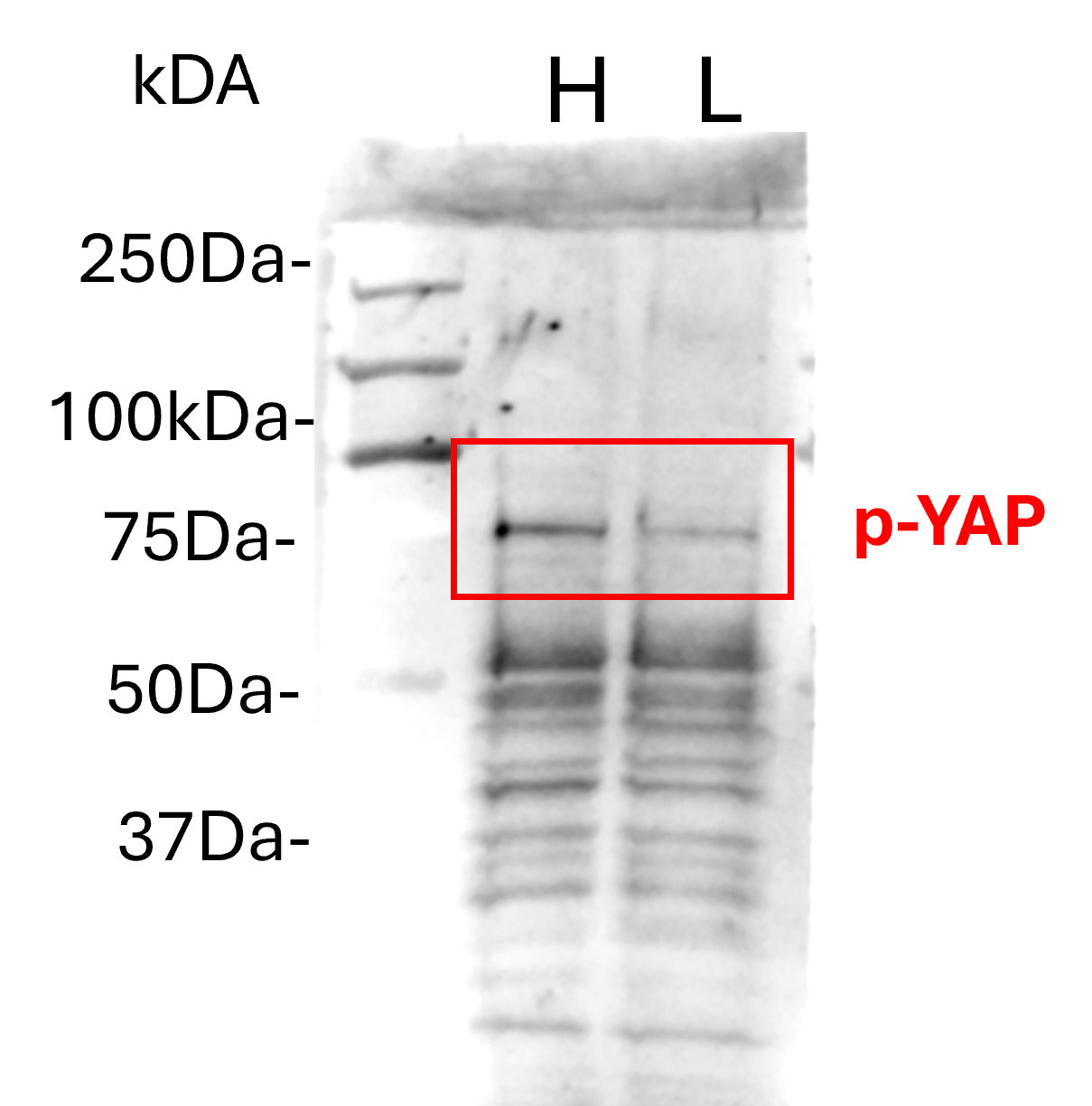

Supplement: Supplementary file 6 — Source data Fig. 4 [file 44318_2025_539_MOESM6_ESM.zip › Figure 4/FIgure 4C/Figure 4C_pYAP.tif]

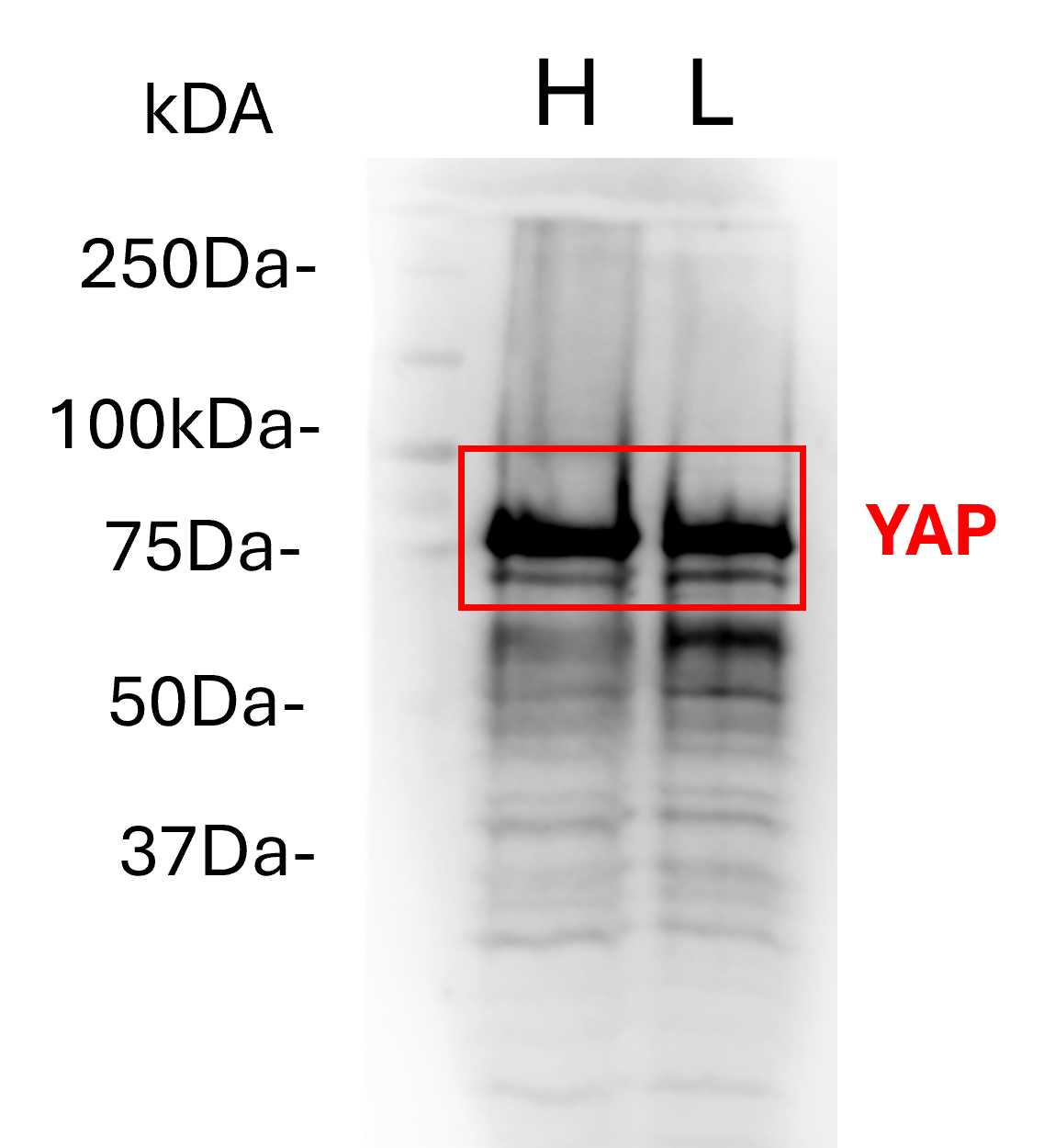

Supplement: Supplementary file 6 — Source data Fig. 4 [file 44318_2025_539_MOESM6_ESM.zip › Figure 4/FIgure 4C/Figure 4C_YAP.tif]

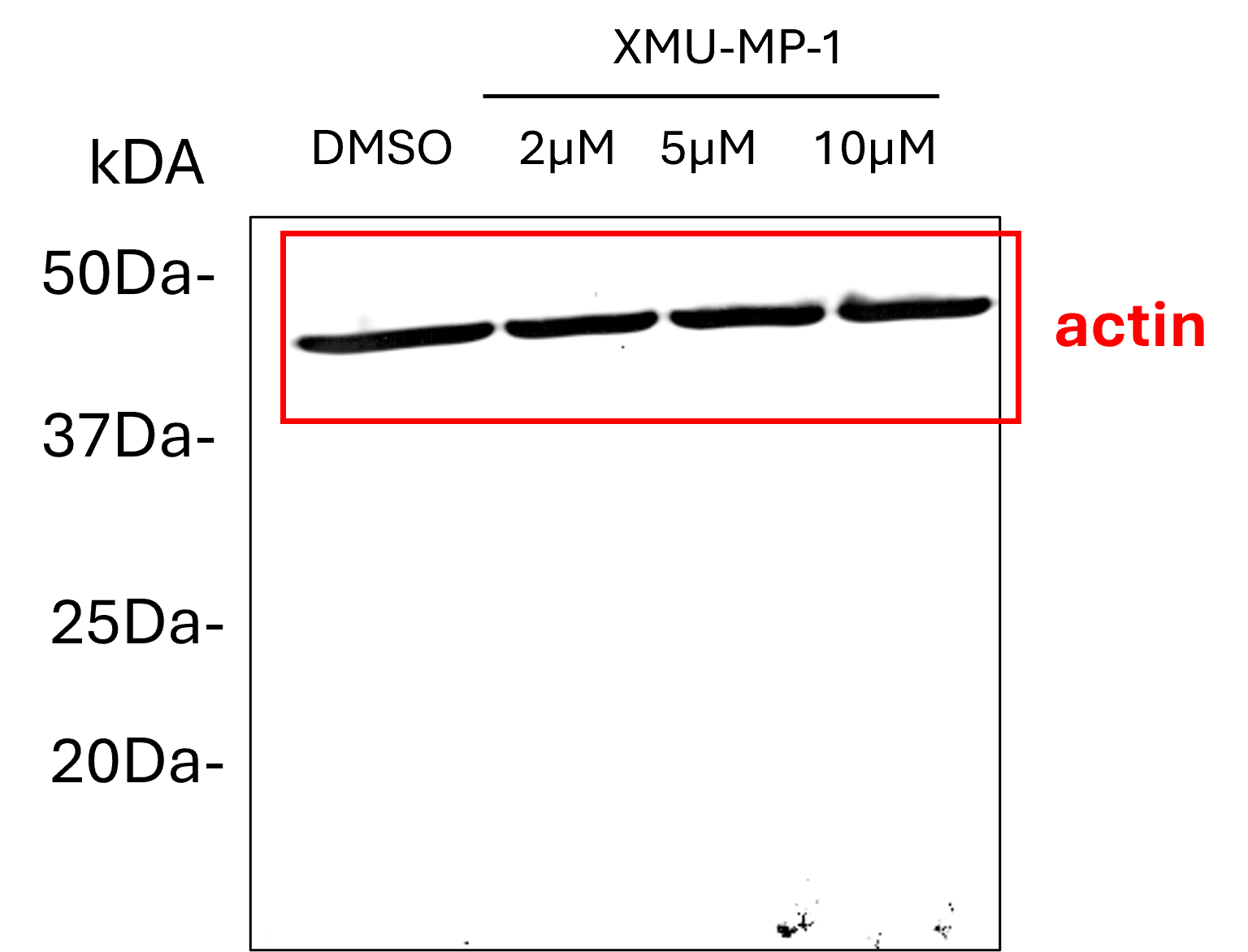

Supplement: Supplementary file 6 — Source data Fig. 4 [file 44318_2025_539_MOESM6_ESM.zip › Figure 4/Figure 4D/actin western.png]

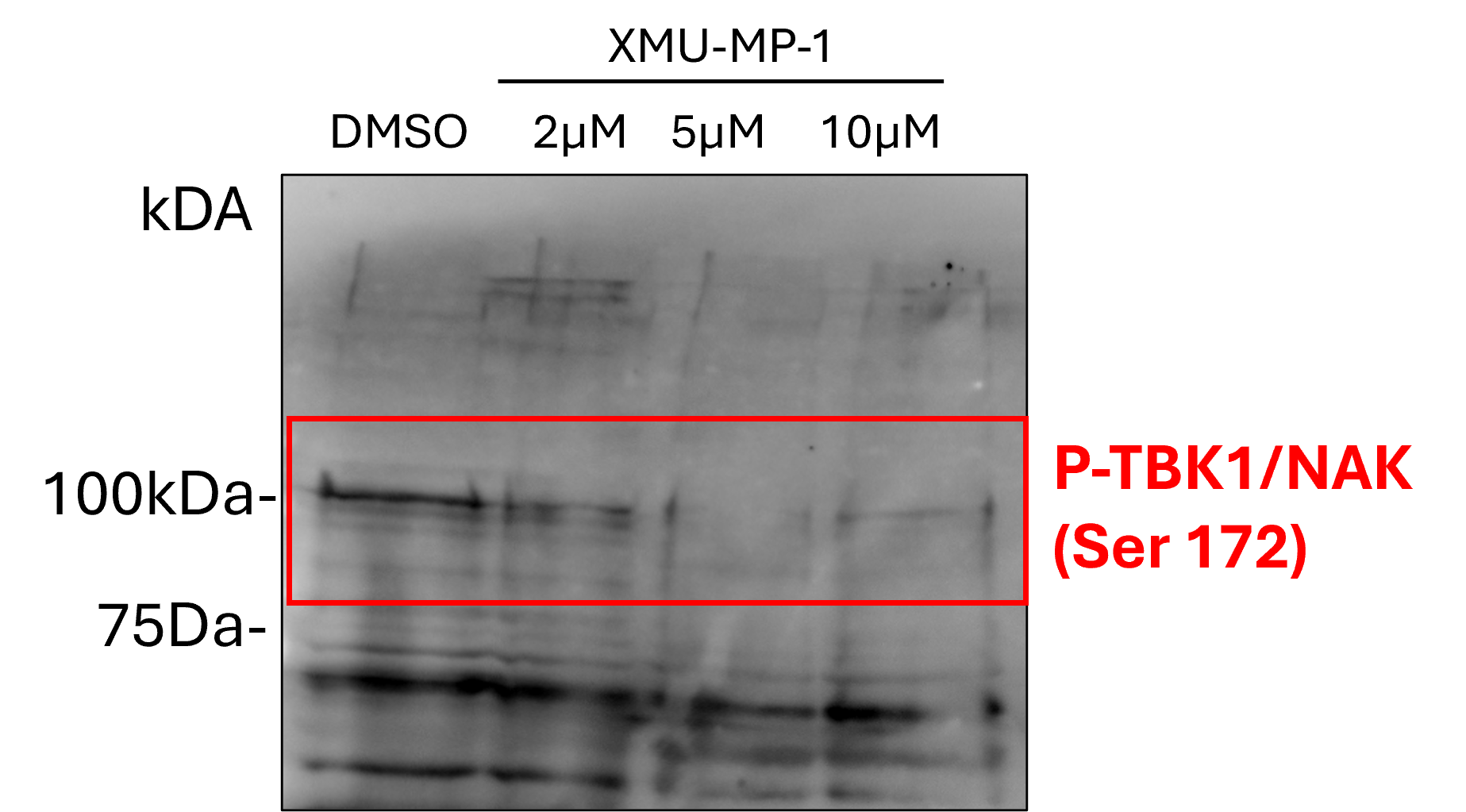

Supplement: Supplementary file 6 — Source data Fig. 4 [file 44318_2025_539_MOESM6_ESM.zip › Figure 4/Figure 4D/p-TBK1 (Ser172) western.png]

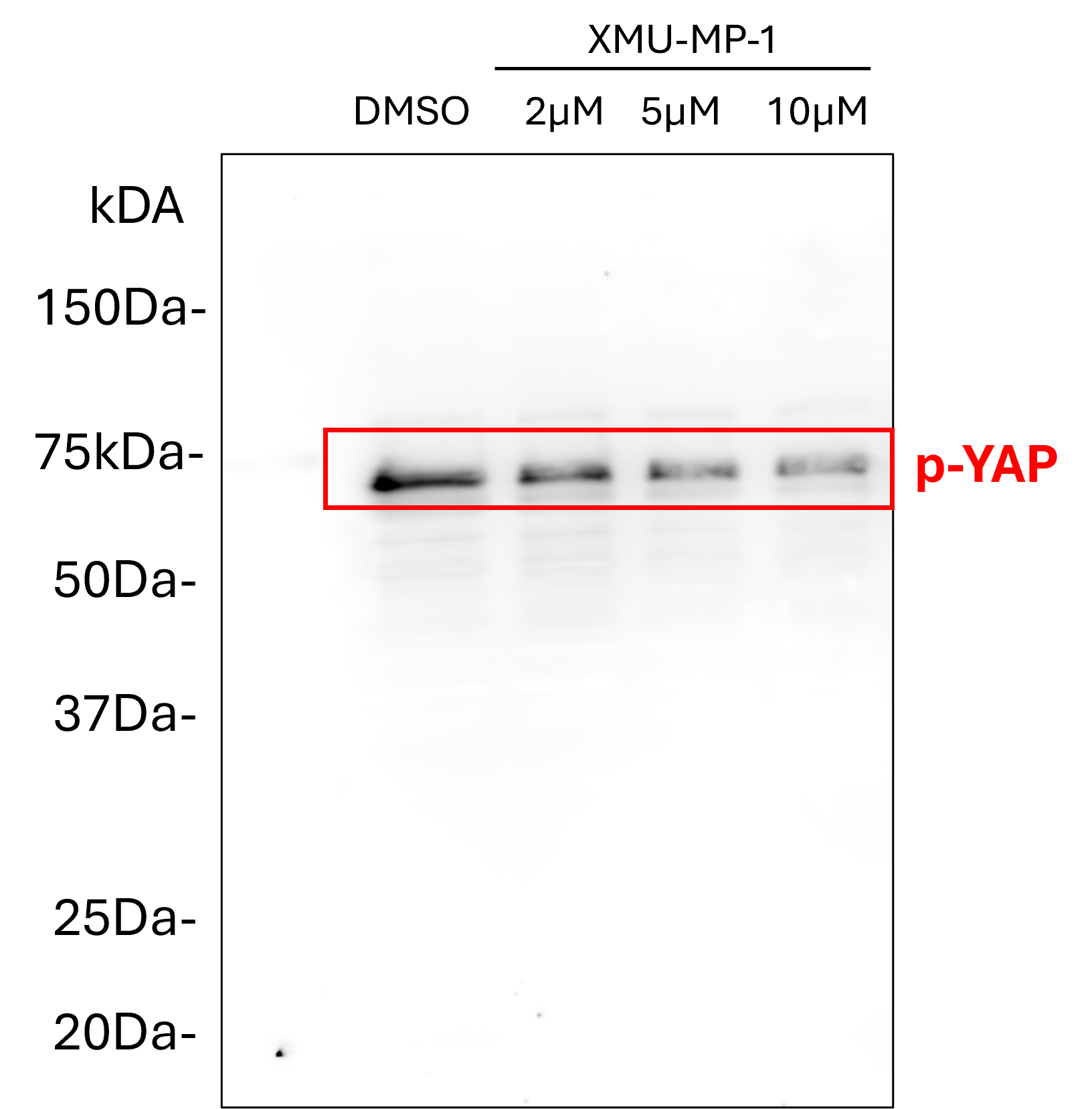

Supplement: Supplementary file 6 — Source data Fig. 4 [file 44318_2025_539_MOESM6_ESM.zip › Figure 4/Figure 4D/p-YAP western.png]

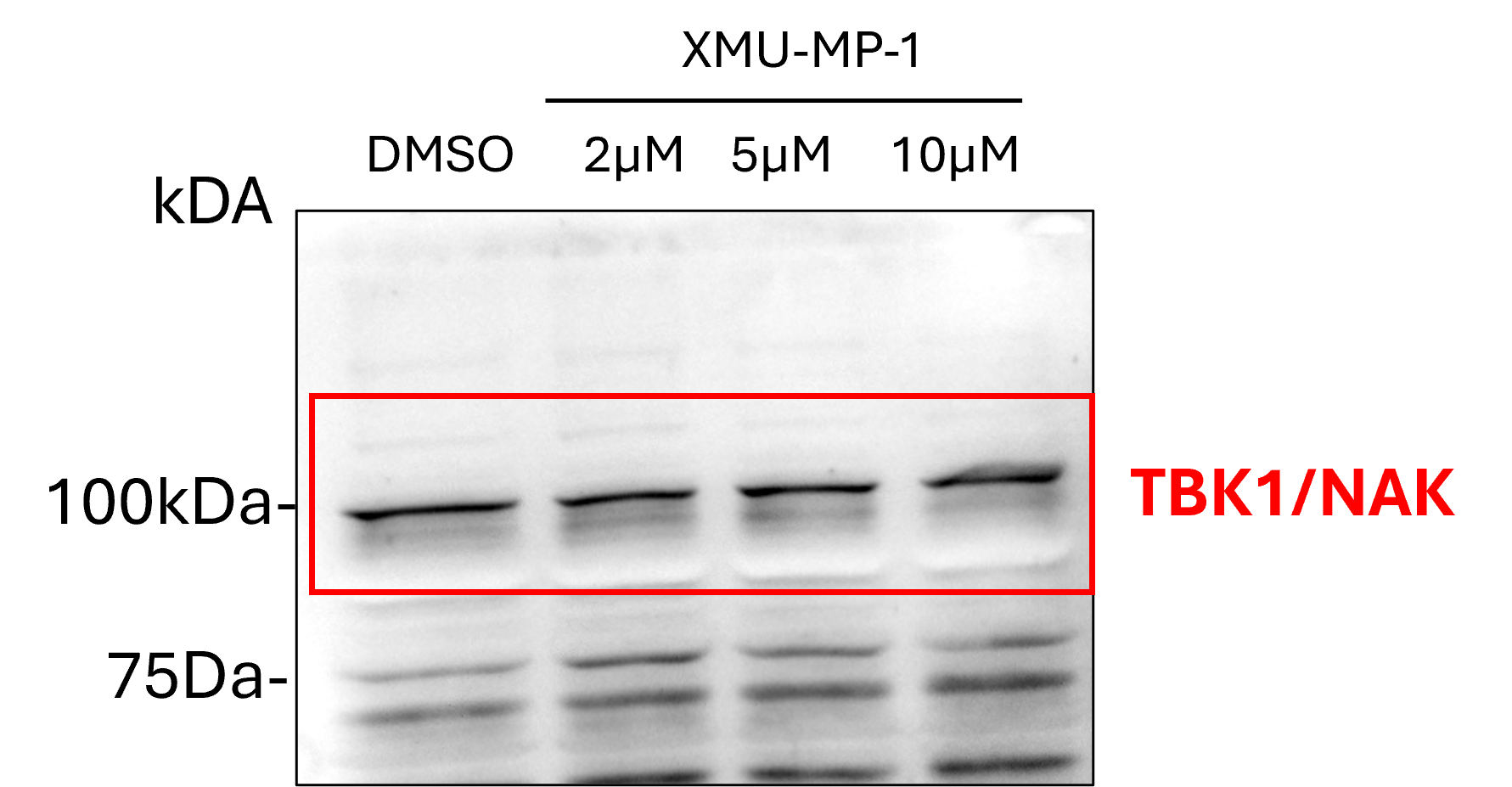

Supplement: Supplementary file 6 — Source data Fig. 4 [file 44318_2025_539_MOESM6_ESM.zip › Figure 4/Figure 4D/TBK1 western.png]

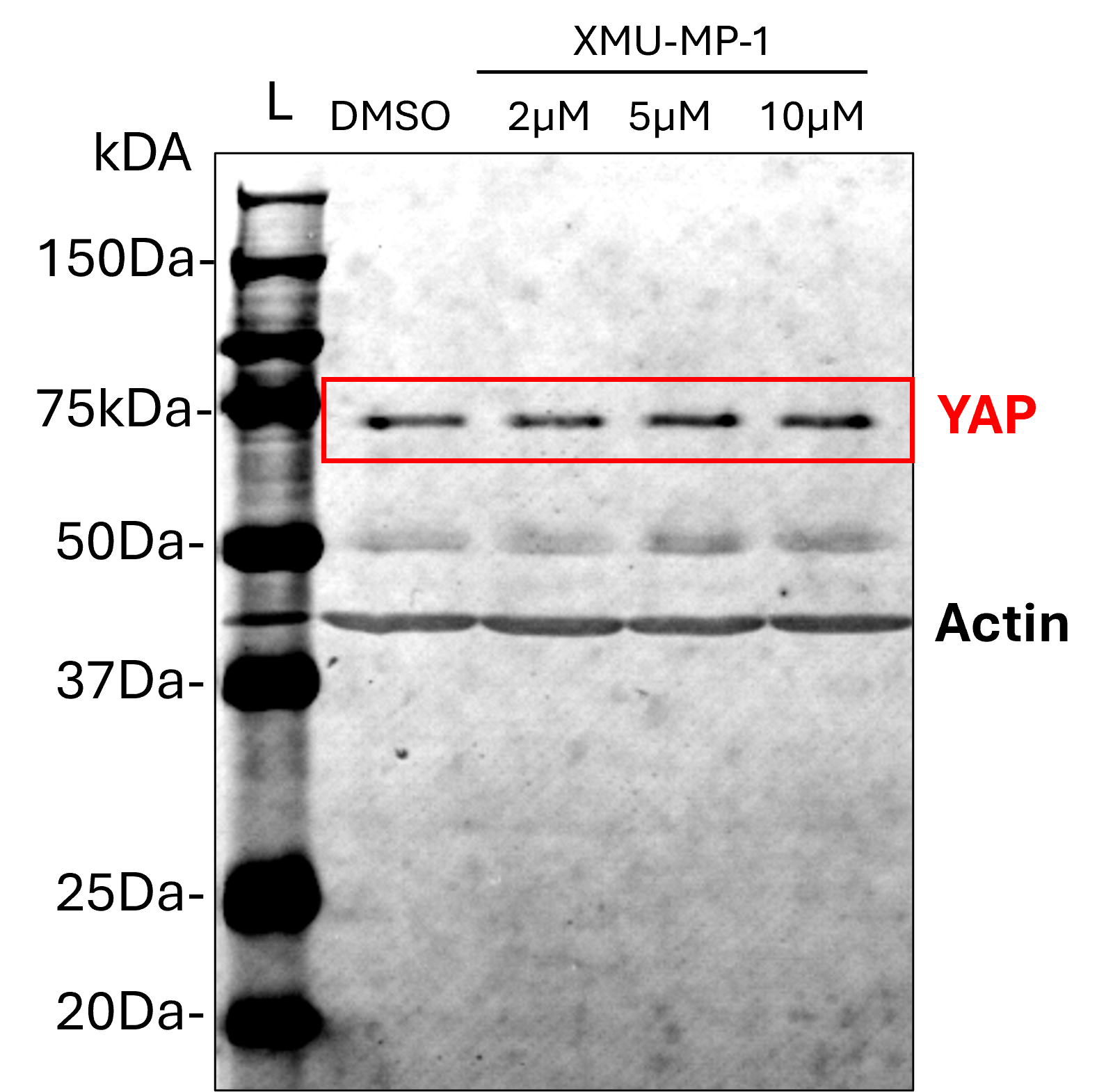

Supplement: Supplementary file 6 — Source data Fig. 4 [file 44318_2025_539_MOESM6_ESM.zip › Figure 4/Figure 4D/YAP western.png]

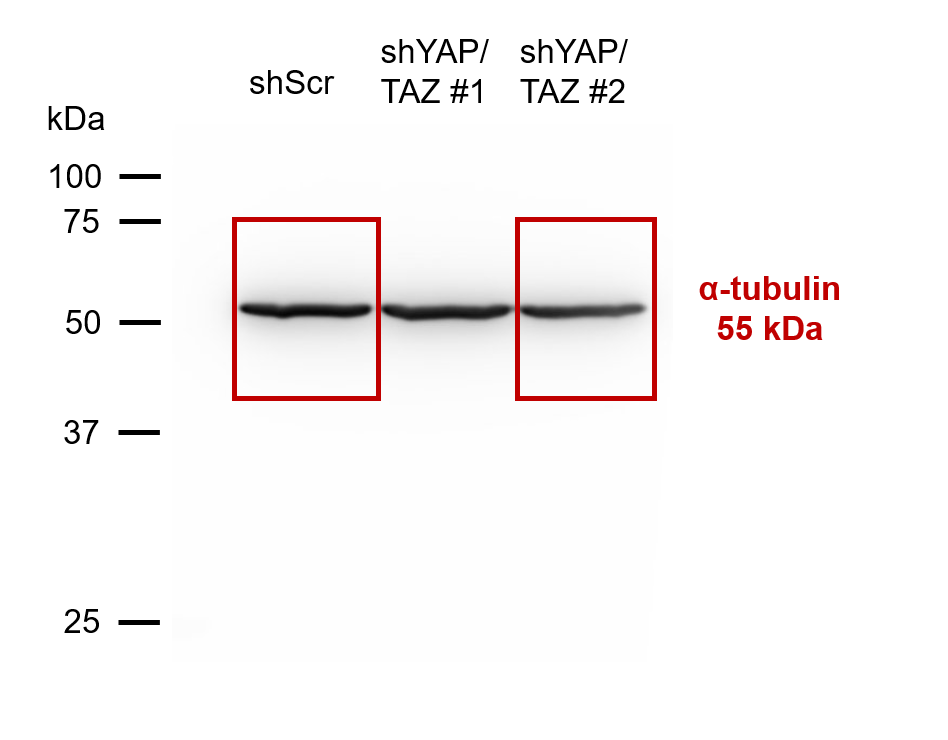

Supplement: Supplementary file 6 — Source data Fig. 4 [file 44318_2025_539_MOESM6_ESM.zip › Figure 4/Figure 4F/Western Tubulin.tif]

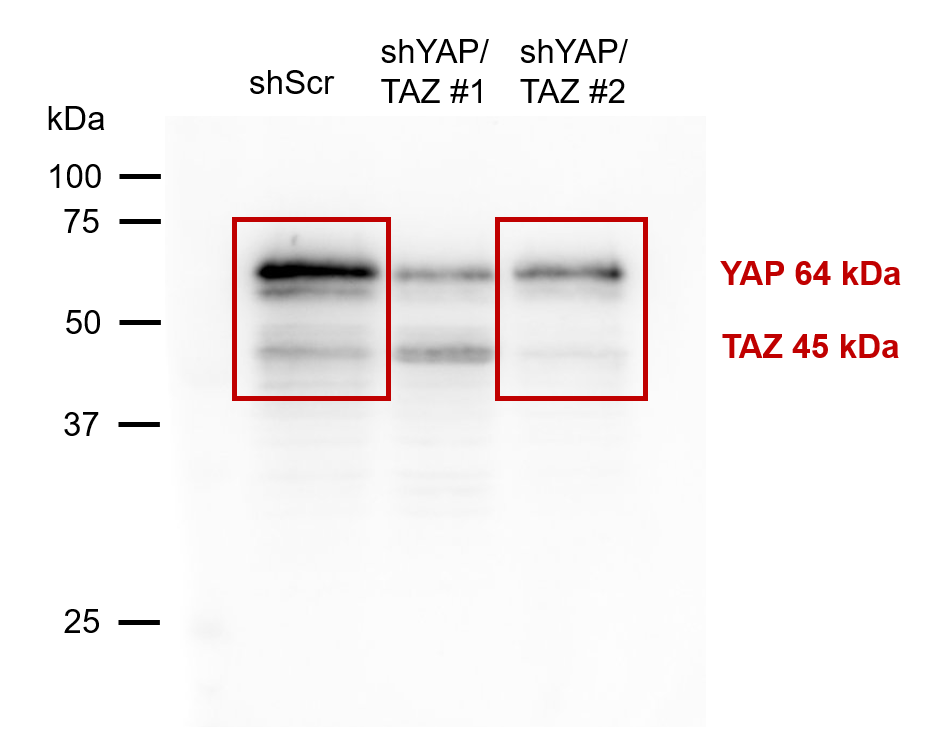

Supplement: Supplementary file 6 — Source data Fig. 4 [file 44318_2025_539_MOESM6_ESM.zip › Figure 4/Figure 4F/Western YAP TAZ.tif]

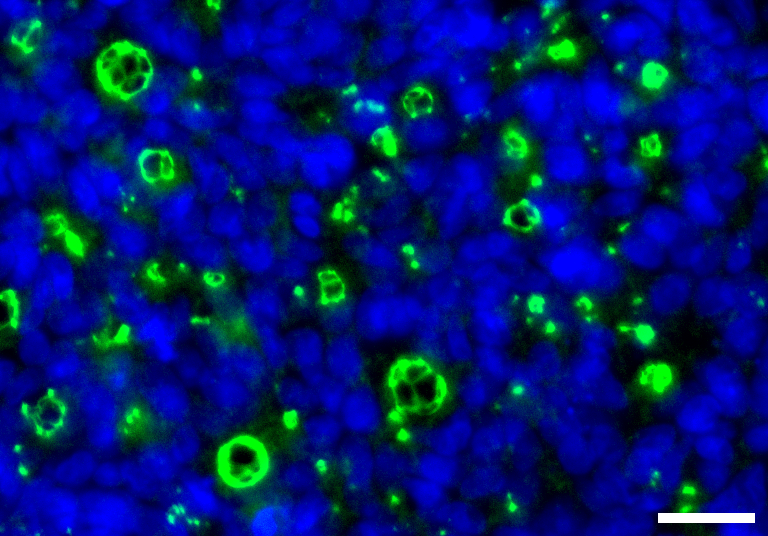

Supplement: Supplementary file 7 — Source data Fig. 5 [file 44318_2025_539_MOESM7_ESM.zip › SD Figure 5/Figure 5A/Figure 5A_IFNL23 KO.tif]

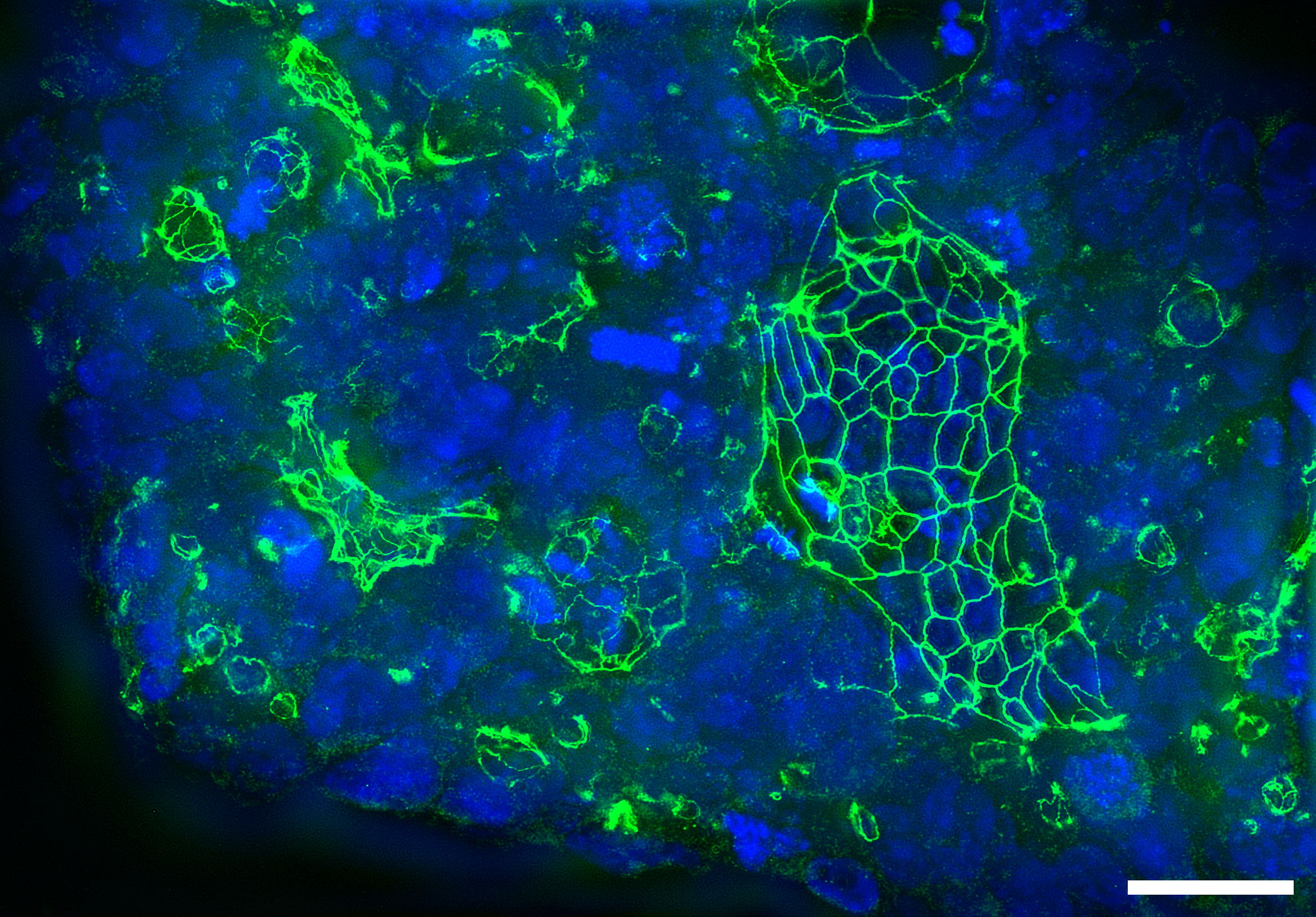

Supplement: Supplementary file 7 — Source data Fig. 5 [file 44318_2025_539_MOESM7_ESM.zip › SD Figure 5/Figure 5A/Figure 5A_IFNL23 KO+0.01ng-ml IFNL23.tif]

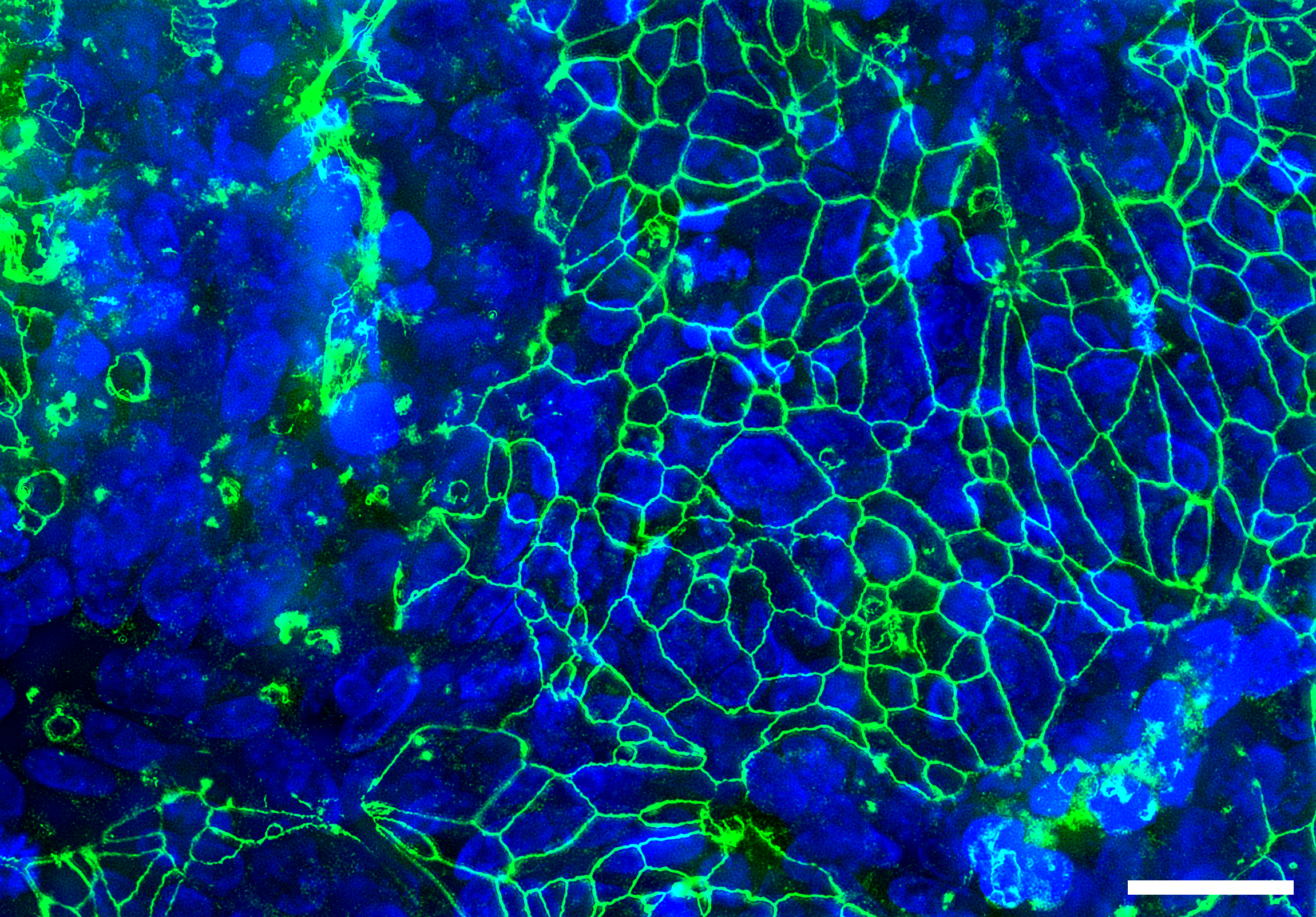

Supplement: Supplementary file 7 — Source data Fig. 5 [file 44318_2025_539_MOESM7_ESM.zip › SD Figure 5/Figure 5A/Figure 5A_IFNL23 KO+100ng-ml IFNL23.tif]

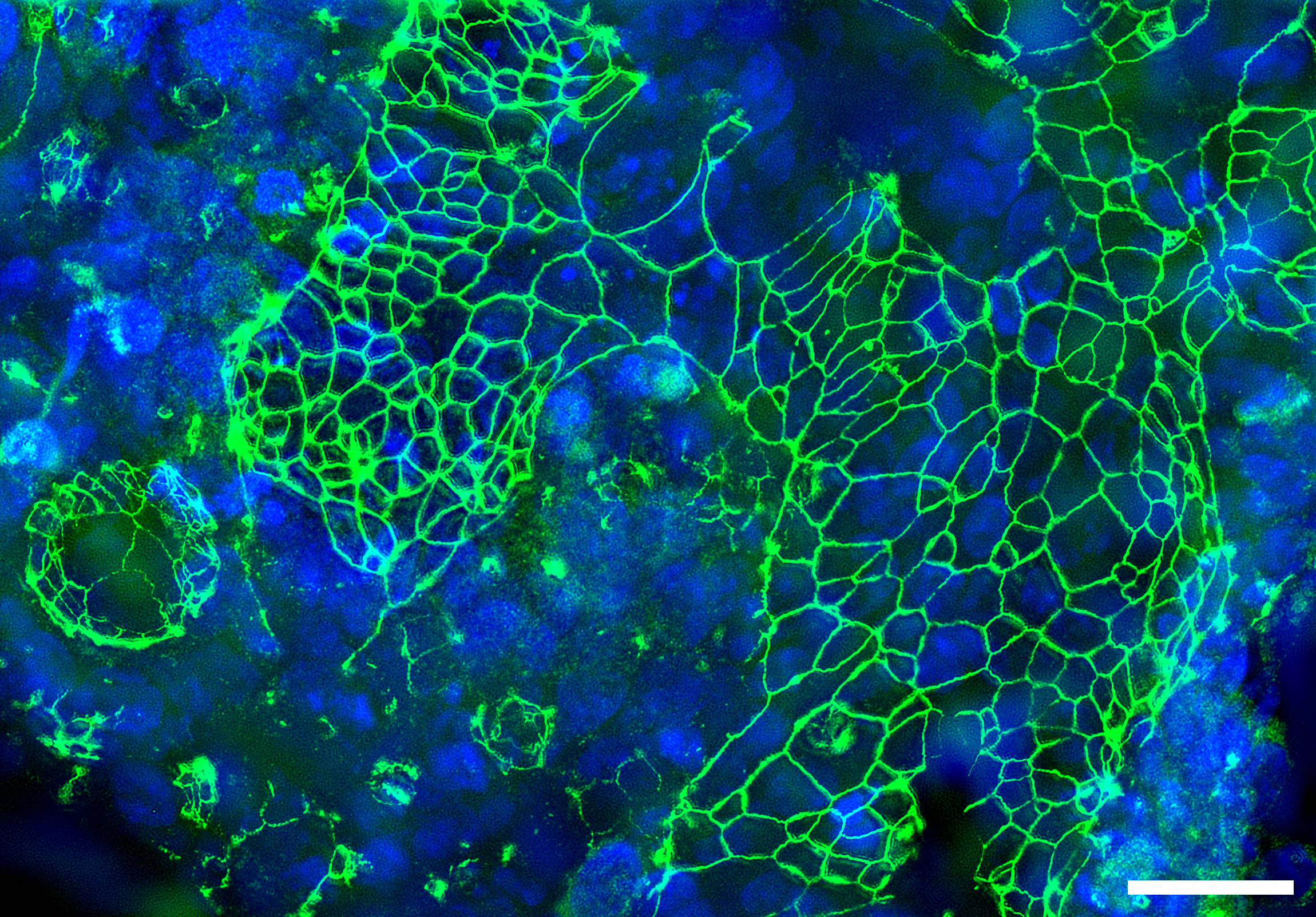

Supplement: Supplementary file 7 — Source data Fig. 5 [file 44318_2025_539_MOESM7_ESM.zip › SD Figure 5/Figure 5A/Figure 5A_IFNL23 KO+1ng-ml IFNL23.tif]

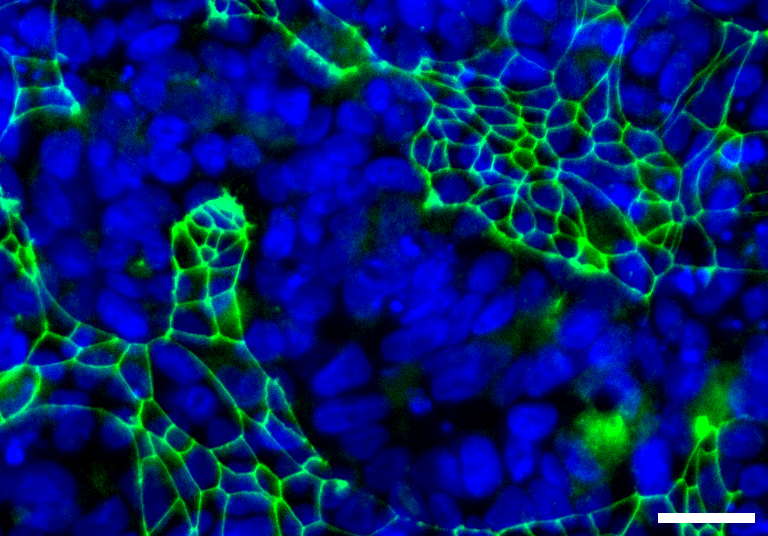

Supplement: Supplementary file 7 — Source data Fig. 5 [file 44318_2025_539_MOESM7_ESM.zip › SD Figure 5/Figure 5A/Figure 5A_IFNLR KO.tif]

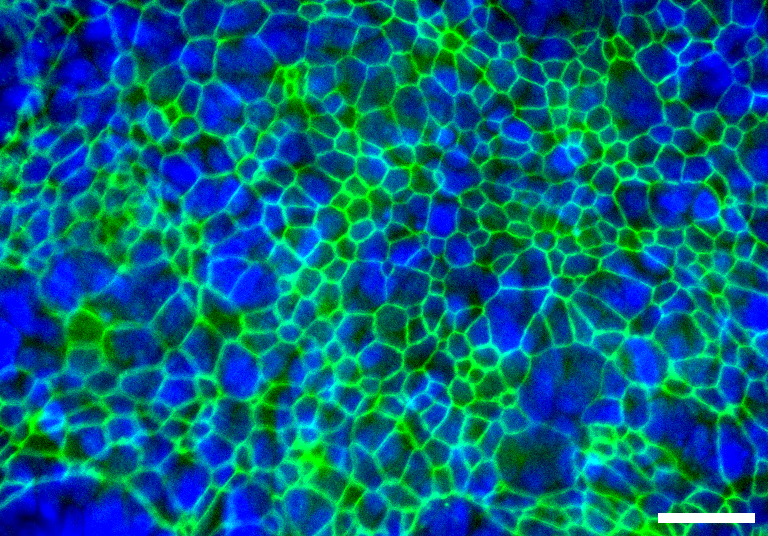

Supplement: Supplementary file 7 — Source data Fig. 5 [file 44318_2025_539_MOESM7_ESM.zip › SD Figure 5/Figure 5A/Figure 5A_WT.tif]

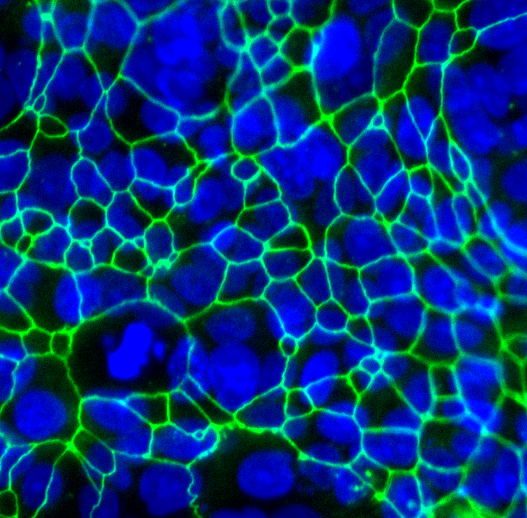

Supplement: Supplementary file 7 — Source data Fig. 5 [file 44318_2025_539_MOESM7_ESM.zip › SD Figure 5/Figure 5F/Figure 5F_DMSO.tif]

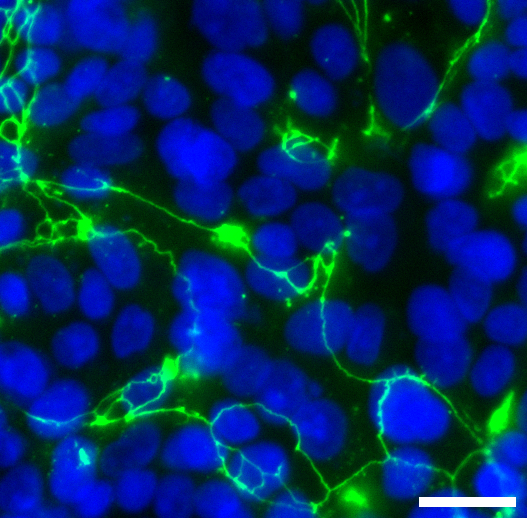

Supplement: Supplementary file 7 — Source data Fig. 5 [file 44318_2025_539_MOESM7_ESM.zip › SD Figure 5/Figure 5F/Figure 5F_H151.tif]

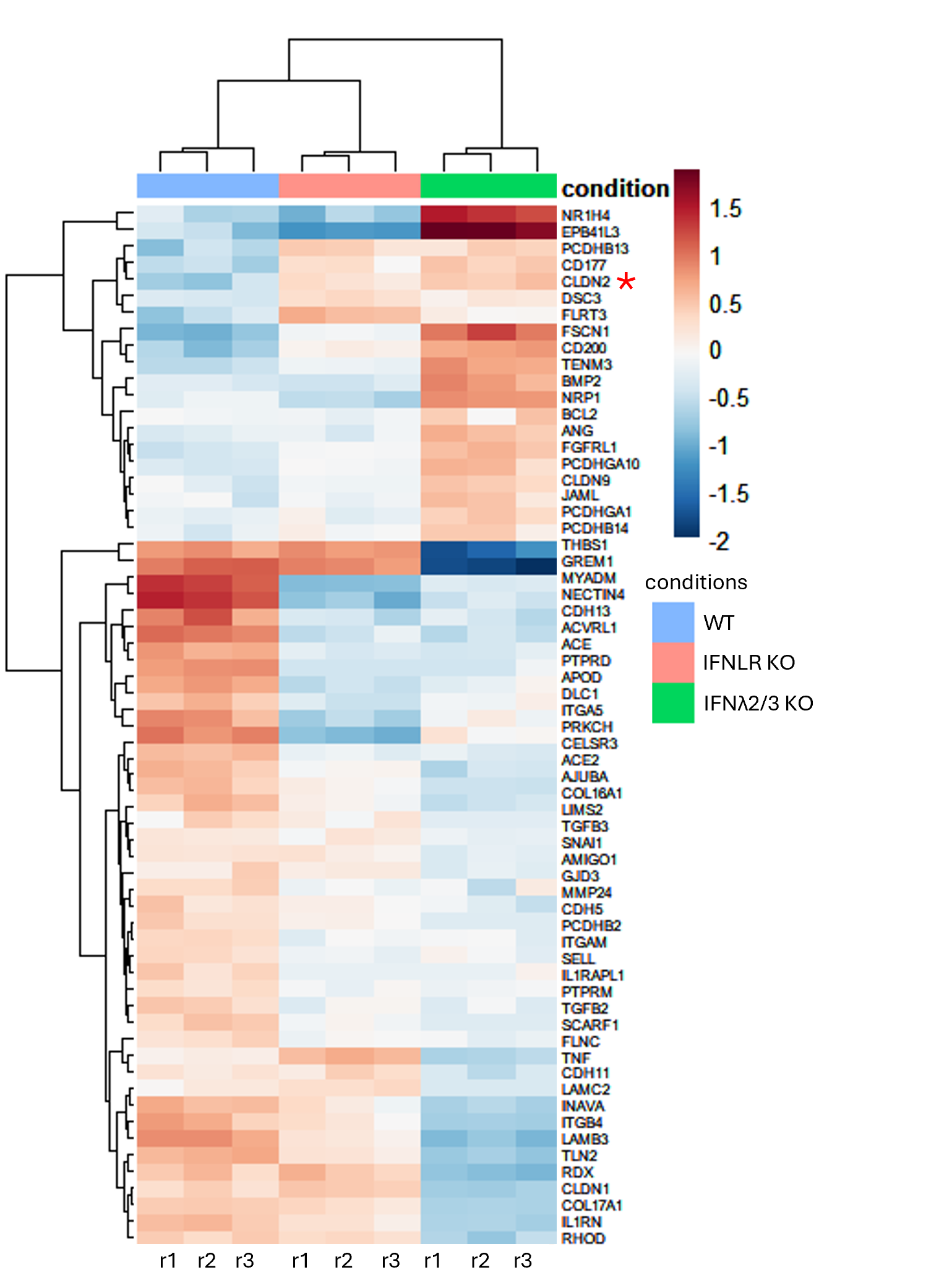

Supplement: Supplementary file 8 — Source data Fig. 6 [file 44318_2025_539_MOESM8_ESM.zip › Figure 6/Figure 6A/Figure 6A.tif]

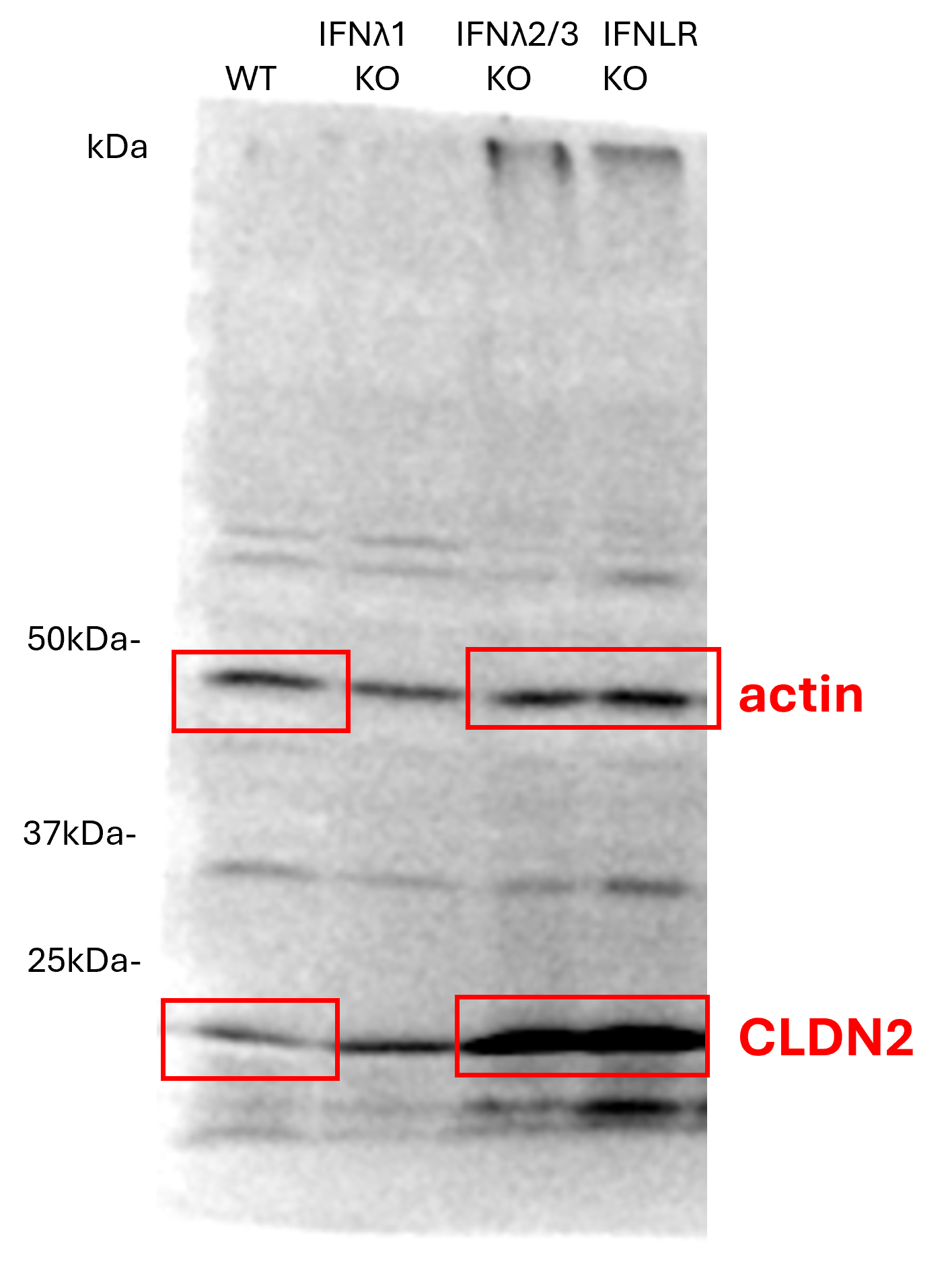

Supplement: Supplementary file 8 — Source data Fig. 6 [file 44318_2025_539_MOESM8_ESM.zip › Figure 6/Figure 6C/Figure 6C.tif]

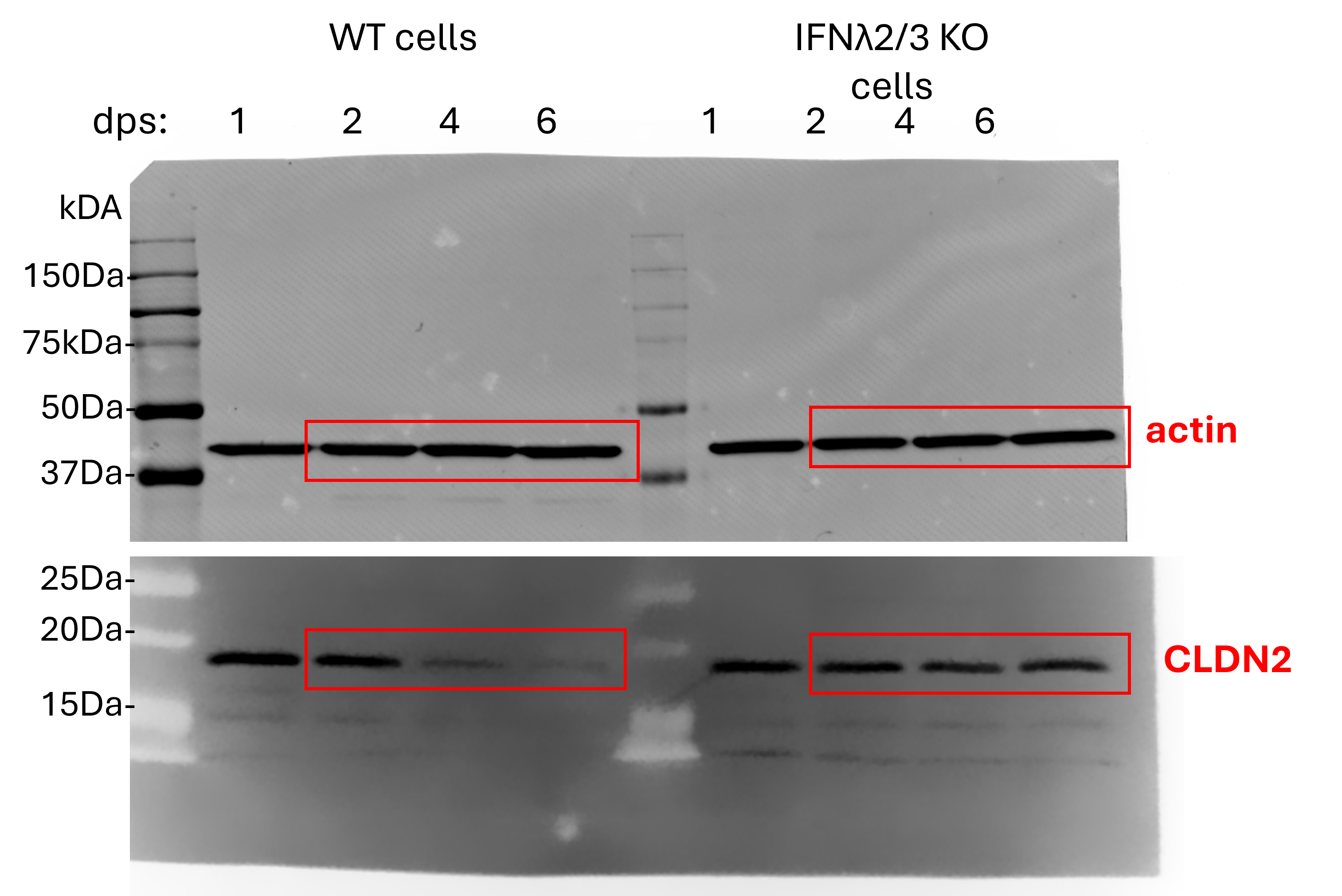

Supplement: Supplementary file 8 — Source data Fig. 6 [file 44318_2025_539_MOESM8_ESM.zip › Figure 6/Figure 6D/Figure 6D.tif]

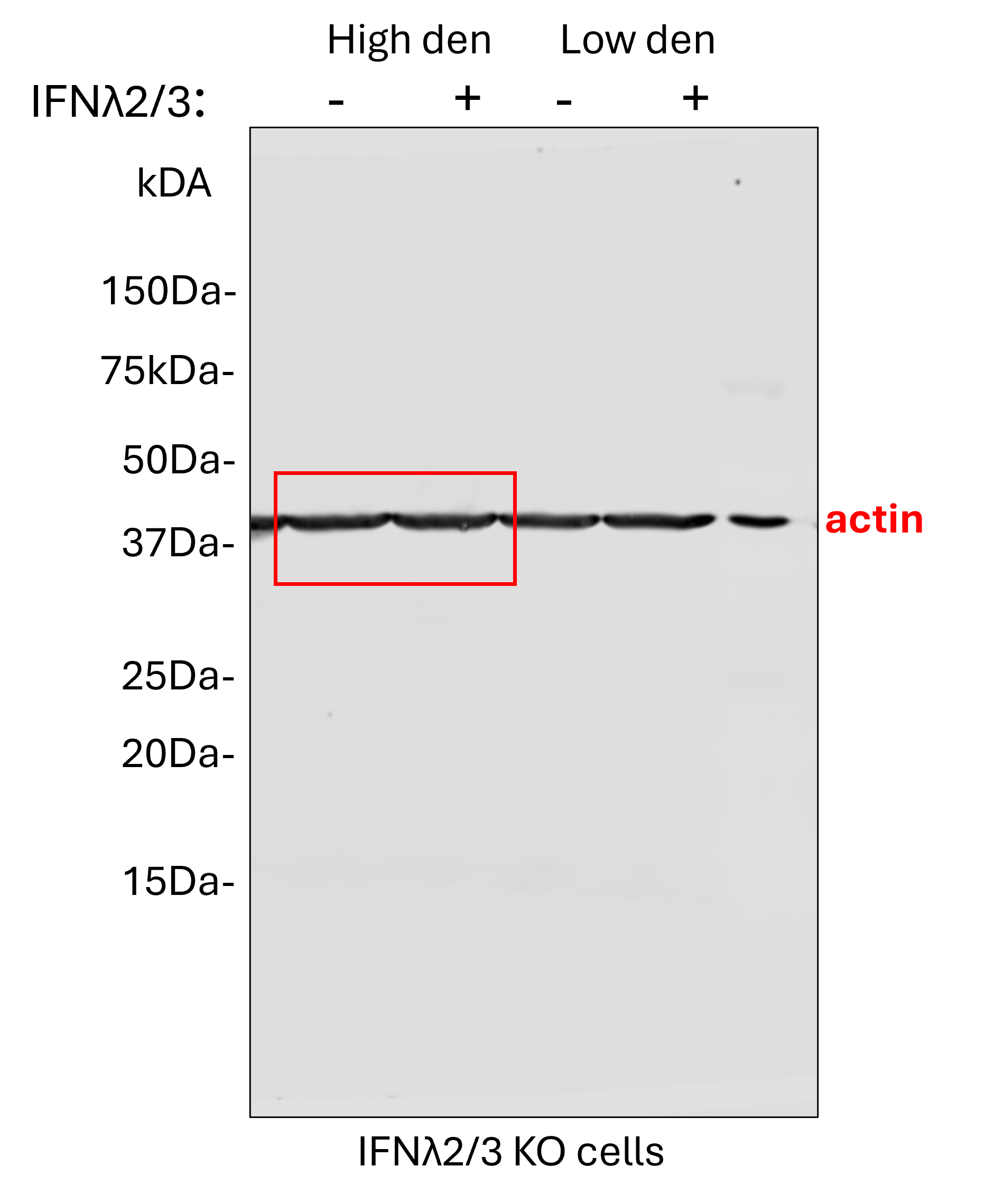

Supplement: Supplementary file 8 — Source data Fig. 6 [file 44318_2025_539_MOESM8_ESM.zip › Figure 6/Figure 6F/IFNL23KO cells_actin_western.tif]

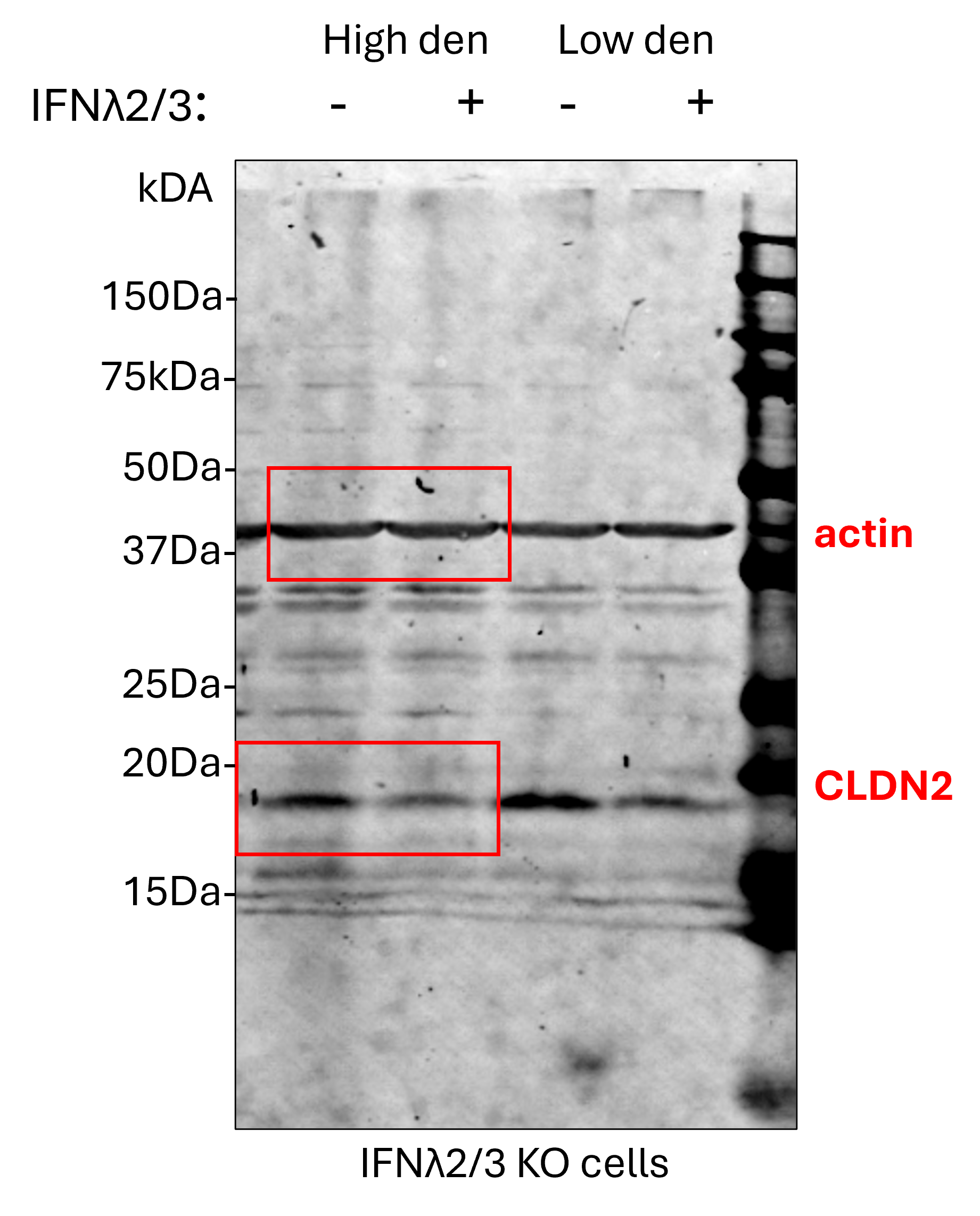

Supplement: Supplementary file 8 — Source data Fig. 6 [file 44318_2025_539_MOESM8_ESM.zip › Figure 6/Figure 6F/IFNL23KO cells_CLDN2_western.tif]

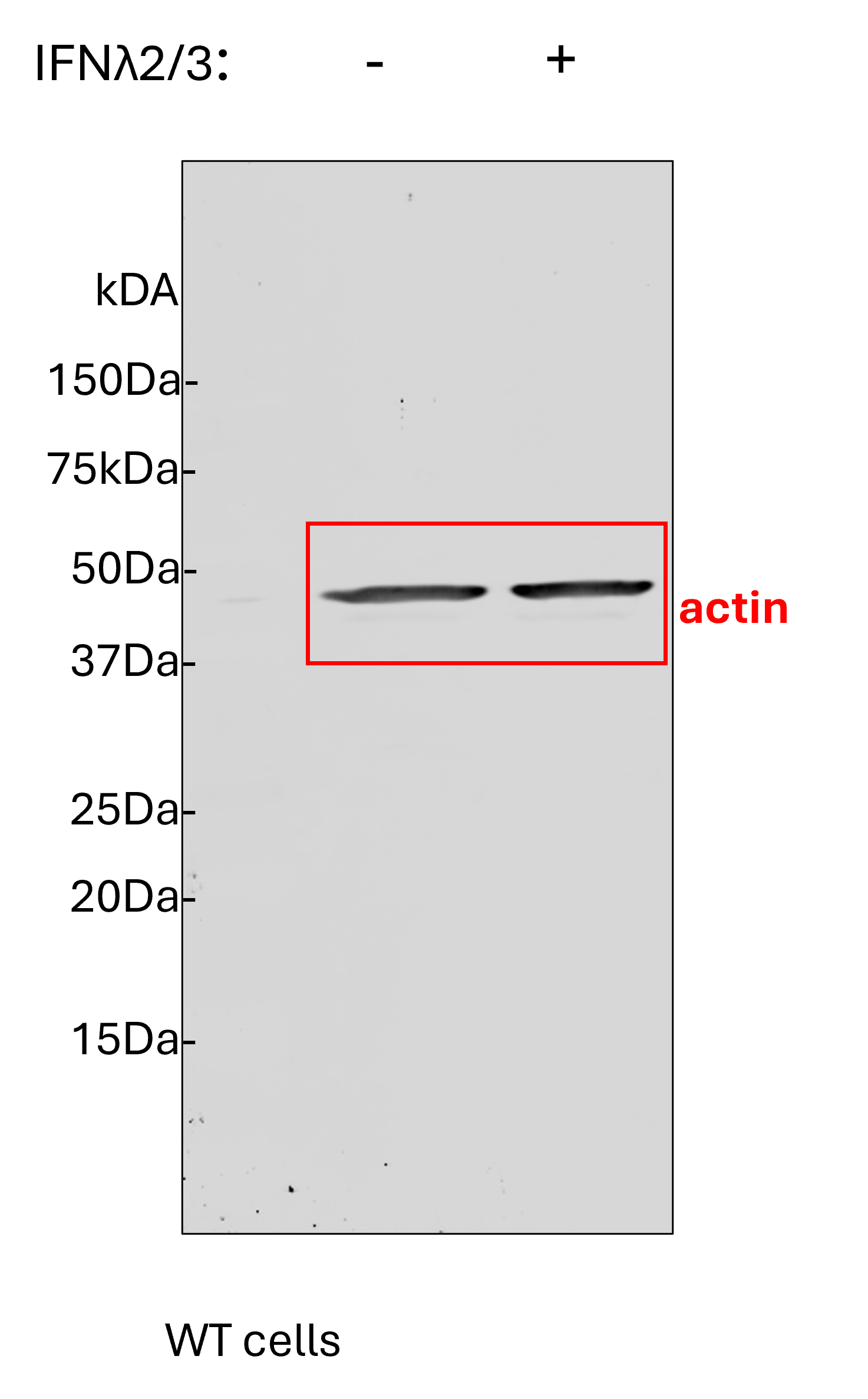

Supplement: Supplementary file 8 — Source data Fig. 6 [file 44318_2025_539_MOESM8_ESM.zip › Figure 6/Figure 6F/WT cells_actin_western.tif]

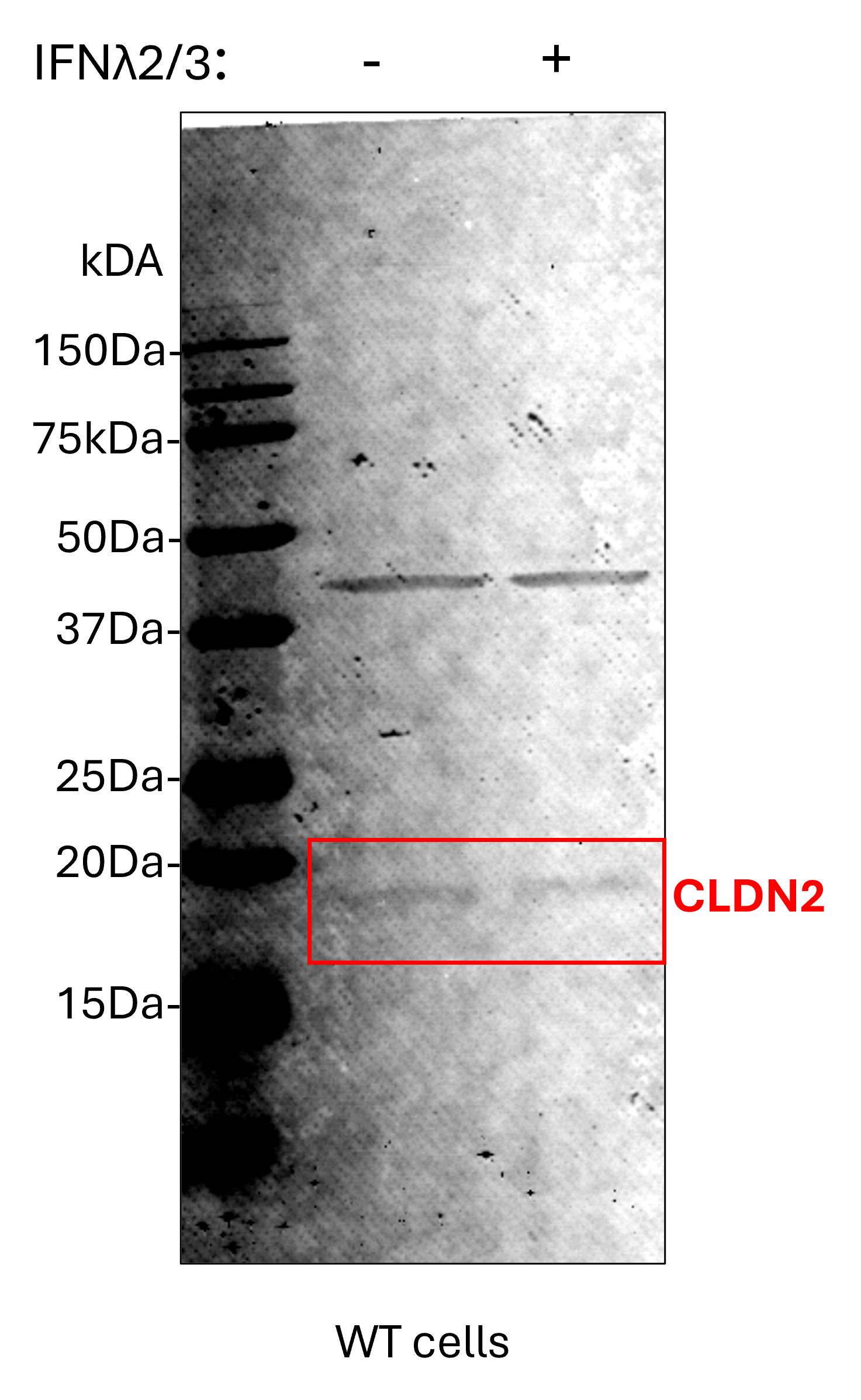

Supplement: Supplementary file 8 — Source data Fig. 6 [file 44318_2025_539_MOESM8_ESM.zip › Figure 6/Figure 6F/WT cells_CLDN2_western.tif]

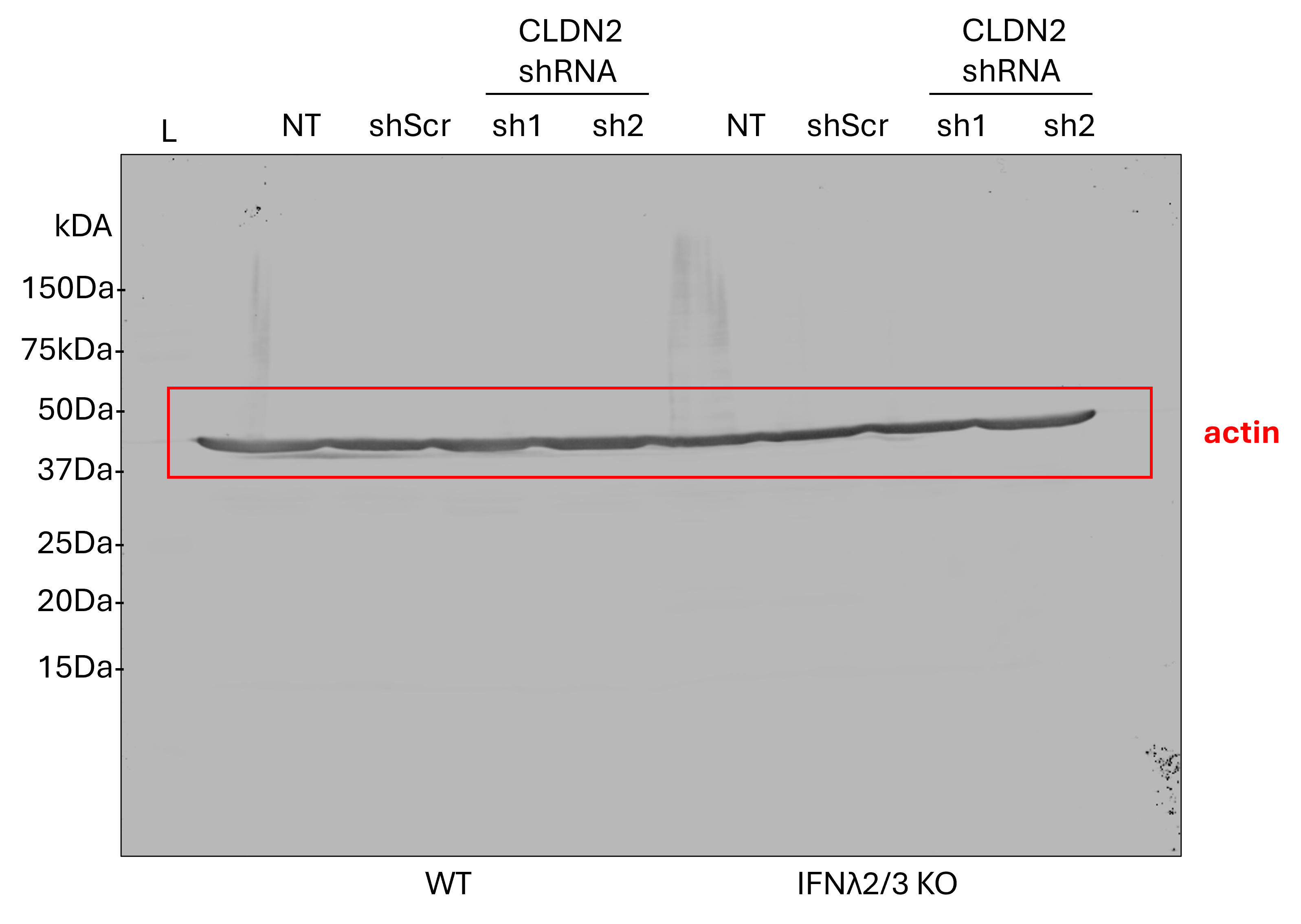

Supplement: Supplementary file 8 — Source data Fig. 6 [file 44318_2025_539_MOESM8_ESM.zip › Figure 6/Figure 6G/Actin_western.tif]

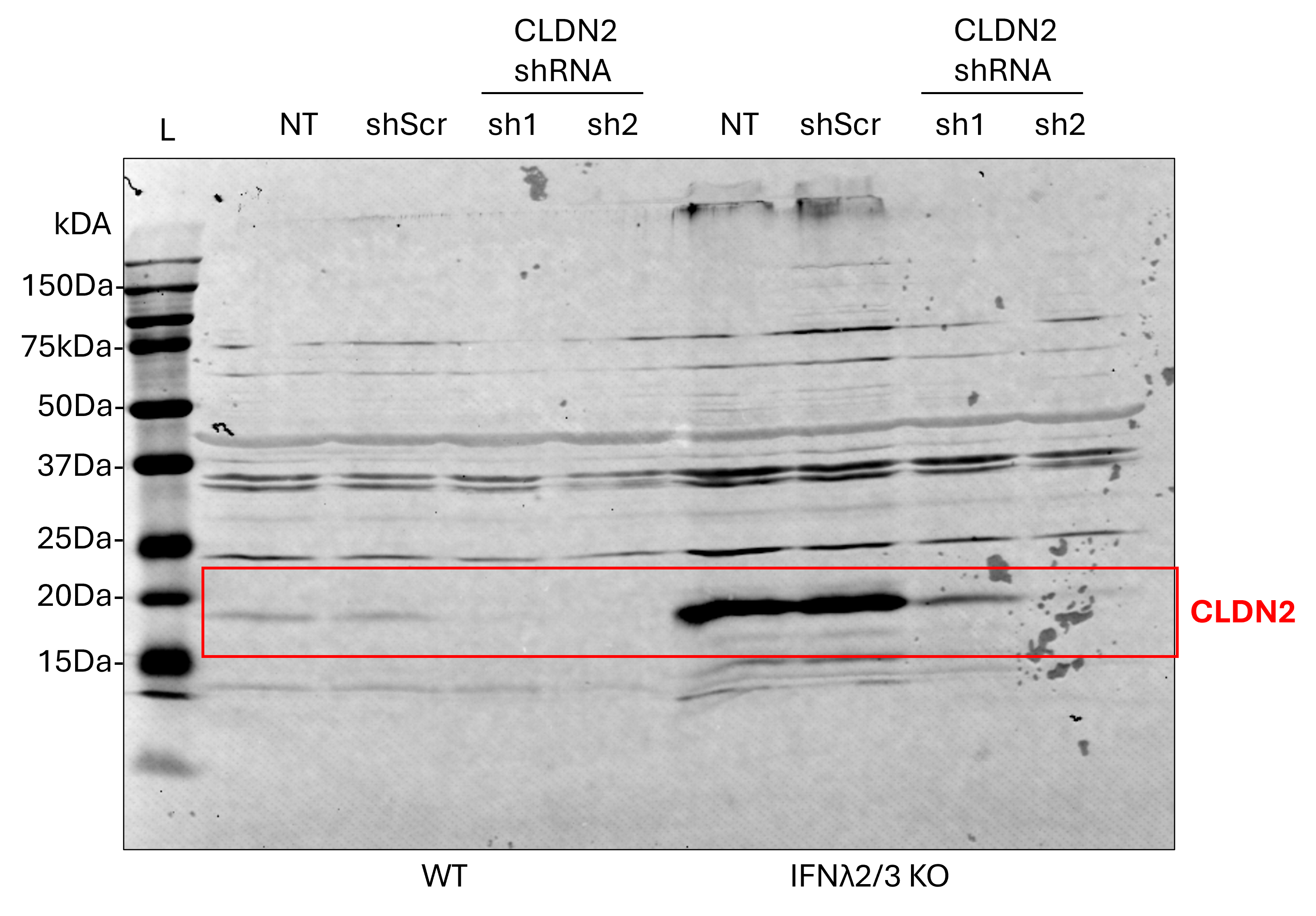

Supplement: Supplementary file 8 — Source data Fig. 6 [file 44318_2025_539_MOESM8_ESM.zip › Figure 6/Figure 6G/CLDN2_western.tif]

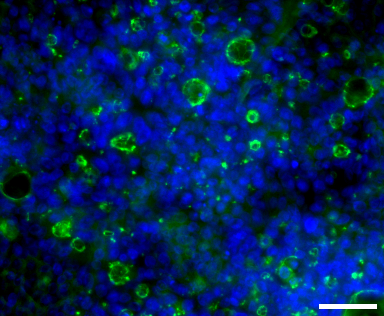

Supplement: Supplementary file 8 — Source data Fig. 6 [file 44318_2025_539_MOESM8_ESM.zip › Figure 6/Figure 6I/Figure 6I_NT.tif]

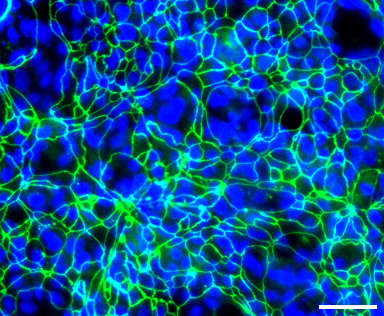

Supplement: Supplementary file 8 — Source data Fig. 6 [file 44318_2025_539_MOESM8_ESM.zip › Figure 6/Figure 6I/Figure 6I_shCLDN1#1.tif]

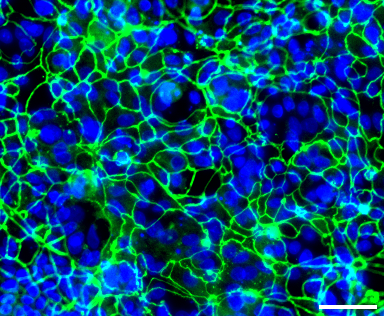

Supplement: Supplementary file 8 — Source data Fig. 6 [file 44318_2025_539_MOESM8_ESM.zip › Figure 6/Figure 6I/Figure 6I_shCLDN1#2.tif]

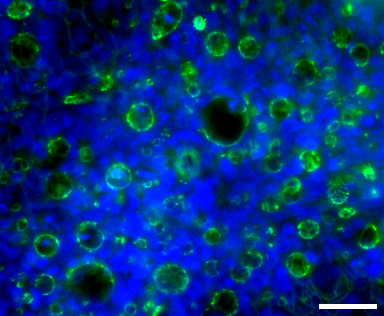

Supplement: Supplementary file 8 — Source data Fig. 6 [file 44318_2025_539_MOESM8_ESM.zip › Figure 6/Figure 6I/Figure 6I_shScr.tif]

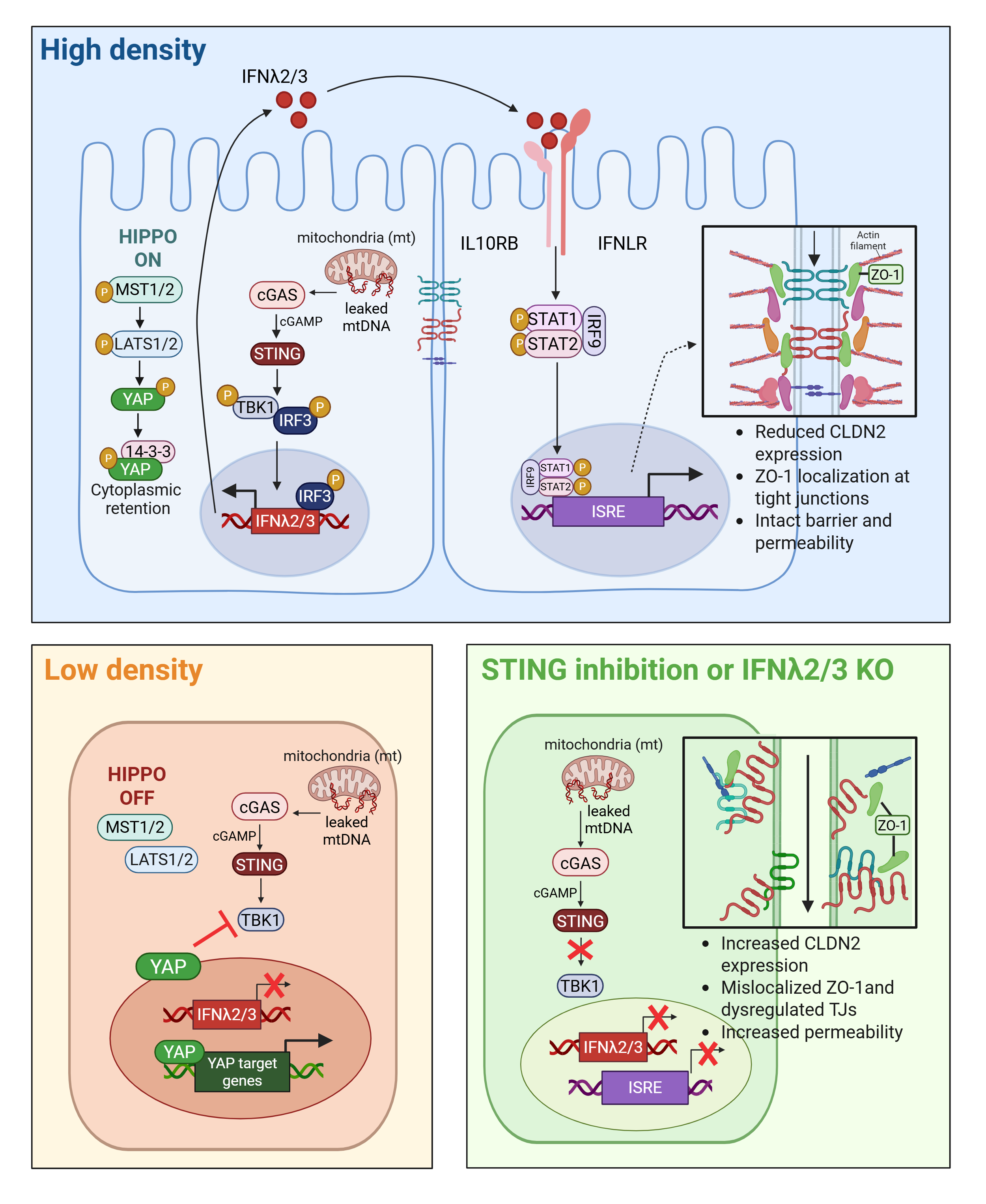

Supplement: Supplementary file 9 — Source data Fig. 7 [file 44318_2025_539_MOESM9_ESM.zip › Figure 7/Figure 7.tif]

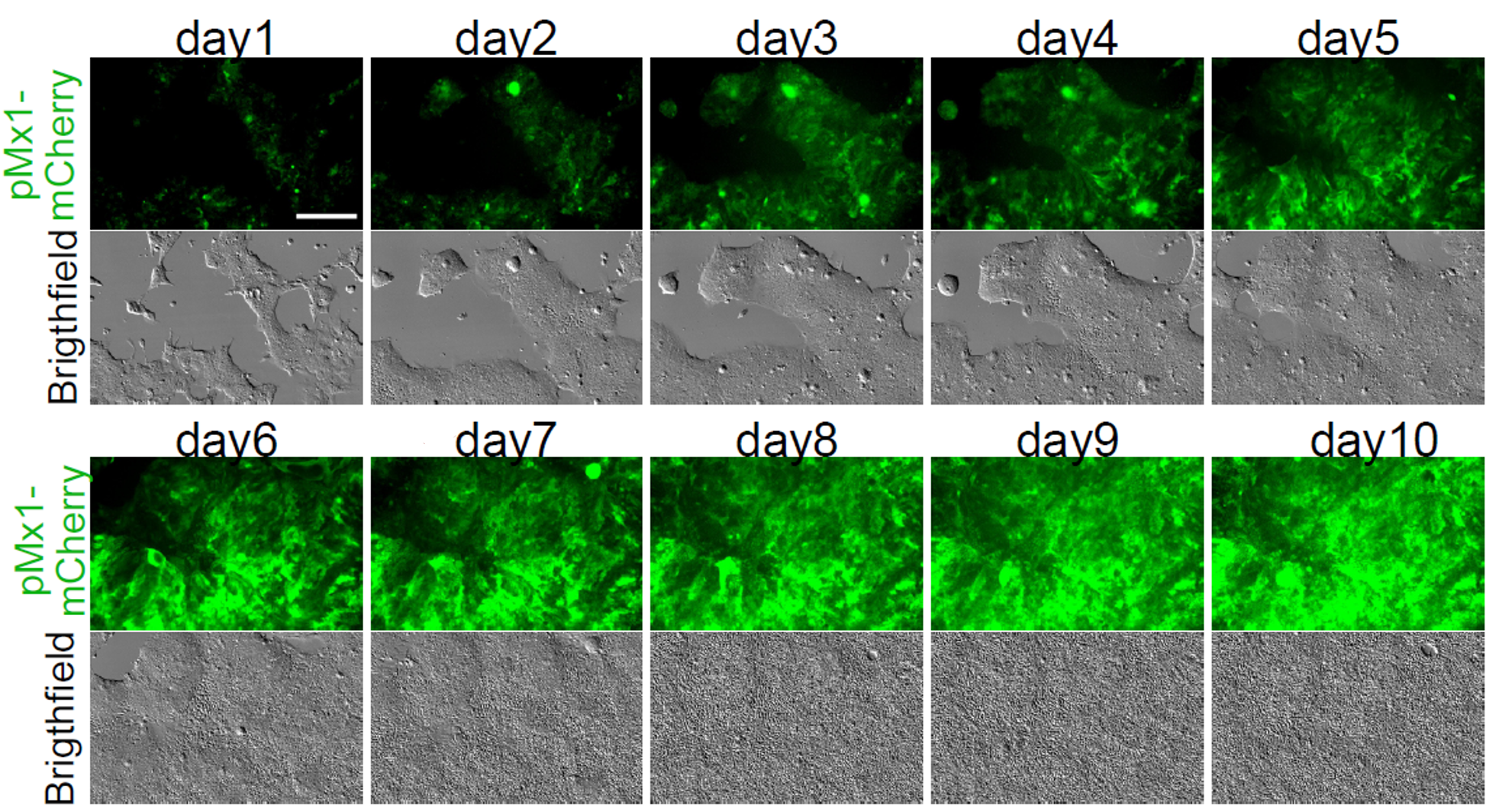

Supplement: Supplementary file 10 — EV Figure Source Data [file 44318_2025_539_MOESM10_ESM.zip › Figure EVs/Figure EV1/Figure EV1B.tif]

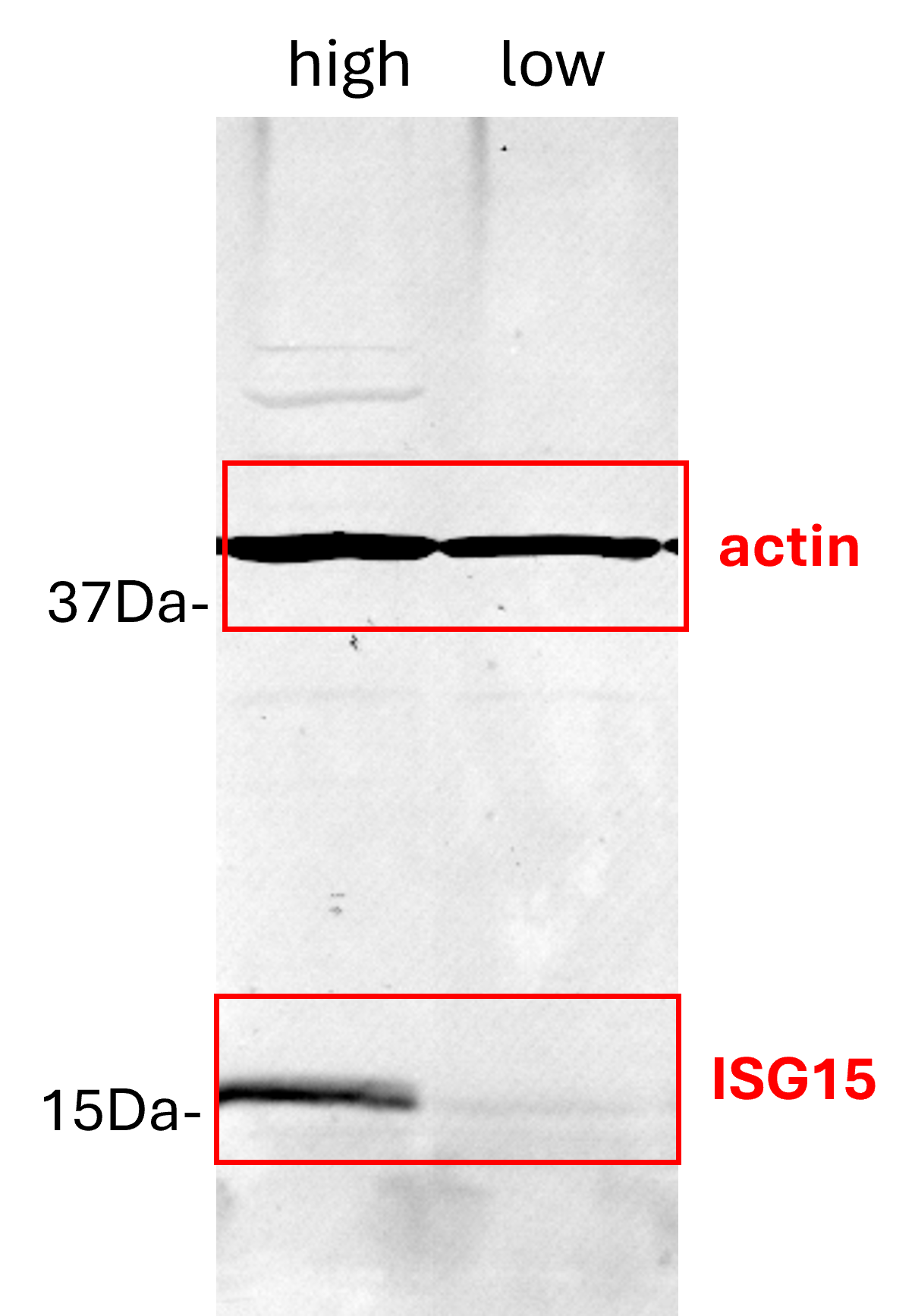

Supplement: Supplementary file 10 — EV Figure Source Data [file 44318_2025_539_MOESM10_ESM.zip › Figure EVs/Figure EV1/Figure EV1F.tif]

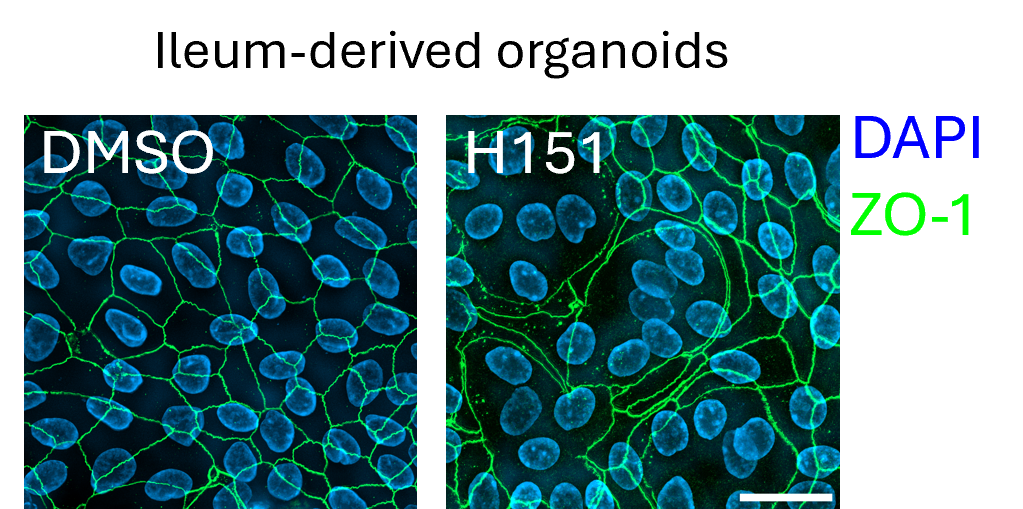

Supplement: Supplementary file 10 — EV Figure Source Data [file 44318_2025_539_MOESM10_ESM.zip › Figure EVs/Figure EV4/Figure EV4A.tif]

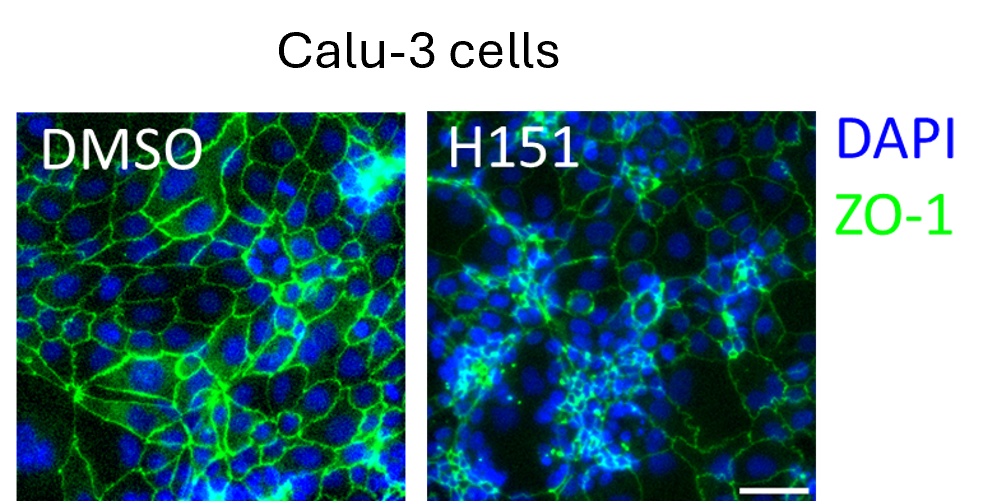

Supplement: Supplementary file 10 — EV Figure Source Data [file 44318_2025_539_MOESM10_ESM.zip › Figure EVs/Figure EV4/Figure EV4B.tif]

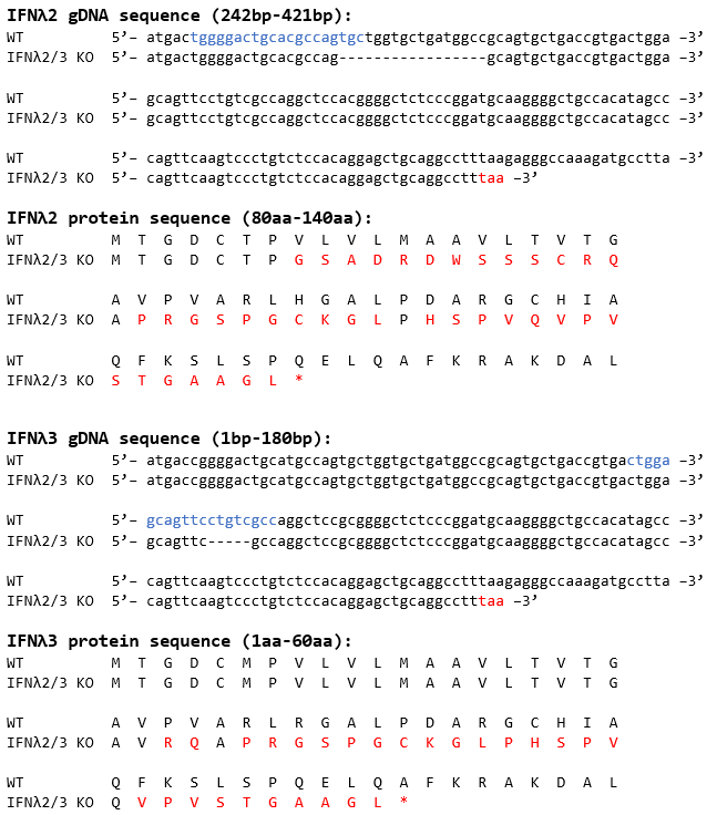

Supplement: Supplementary file 11 — Appendix Source Data [file 44318_2025_539_MOESM11_ESM.zip › Appendix Figures/Appendix Figure S1/Appendix Figure S1.tif]
