# Supplementary material for: Basal IFN-λ2/3 expression mediates tight junction formation in human epithelial cells
Source: EMBO J. 2025 Sep 1;44(20):5785–815. doi: 10.1038/s44318-025-00539-5 (PMC12528397; doi:10.1038/s44318-025-00539-5)
Supplement: Supplementary file 12 — Expanded View Figures [file 44318_2025_539_MOESM12_ESM.pdf]

Expanded View Figures

Figure EV1. Cell density regulates the basal immune response in human intestinal and airway epithelial cells.

(**A**) T84 cells seeded on transwells were harvested at indicated days post-seeding and the expression of the ISGs Mx1, IFIT1, ISG15 and Viperin were assessed by qRT-PCR. (**B–D**) T84 pMx1-mCherry cells were seeded at low cellular density. One-day post-seeding cells were imaged over a period of ten days to visualize the expression of the mCherry reporter whose expression is under the control of the ISG Mx1 promoter region. (**B**) Representative image, Scale bar = 50 µm. (**C**) Quantification of the pMx1-mCherry mean fluorescence intensity over time. (**D**) Positive correlation between cell density and expression of the pMx1-mCherry reporter. Linear regression and coefficient of correlation (r) were calculated using the mean fluorescence intensity from (**C**) and the area occupied by cells. (**E**, **F**) T84 cells were seeded at high and low cellular density. (**E**) Expression of the ISGs Mx1, IFIT1 and Viperin was addressed using qRT-PCR. The relative expression was normalized to TBP. $n \geq 3$ biological replicates. (**F**) ISG15 expression was assessed by Western Blot. ISG15 abundance was quantified relative to the loading control actin. (**G**) The expression of IFN-λ2/3 and of the ISGs Mx1, IFIT1 and Viperin was addressed by qRT-PCR in Calu-3 cells seeded at high and low cellular densities. The relative expression was normalized to TBP. $n \geq 3$ biological replicates. Statistical analysis was performed using ordinary one-way ANOVA using the cells at one day post-seeding as a reference (**A**, **C**) and unpaired *t* test between high and low density (**E**, **G**). n.s. indicates non-significant results ($P > 0.05$). Exact *P* values are shown on the plots when significant; otherwise, results are not significant. Error bars represent standard deviation with the mean as the center. Source data are available online for this figure.

 © The Author(s)

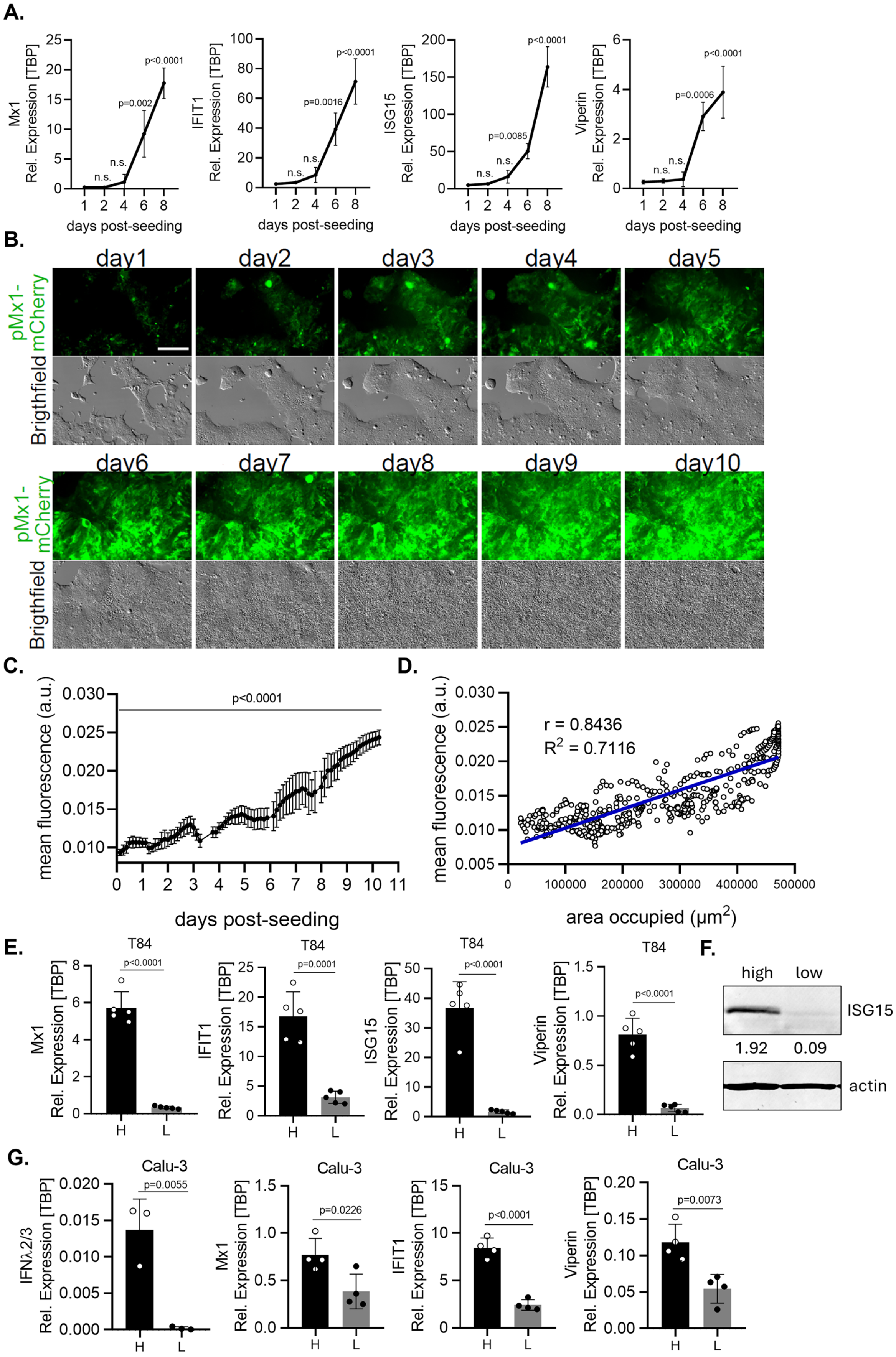
A.
Mx1 Rel. Expression [TBP]
IFIT1 Rel. Expression [TBP]
ISG15 Rel. Expression [TBP]
Viperin Rel. Expression [TBP]
days post-seeding
n.s.
p=0.002
p=0.0016
p=0.0085
p=0.0006
p<0.0001
B.
day1
day2
day3
day4
day5
day6
day7
day8
day9
day10
pMx1-mCherry
Brigthfield
C.
mean fluorescence (a.u.)
p<0.0001
days post-seeding
D.
r = 0.8436
R2 = 0.7116
area occupied (µm2)
E.
T84
p=0.0001
F.
high
low
ISG15
1.92
0.09
actin
G.
Calu-3
IFNλ2/3 Rel. Expression [TBP]
p=0.0055
p=0.0226
p=0.0073
H
L

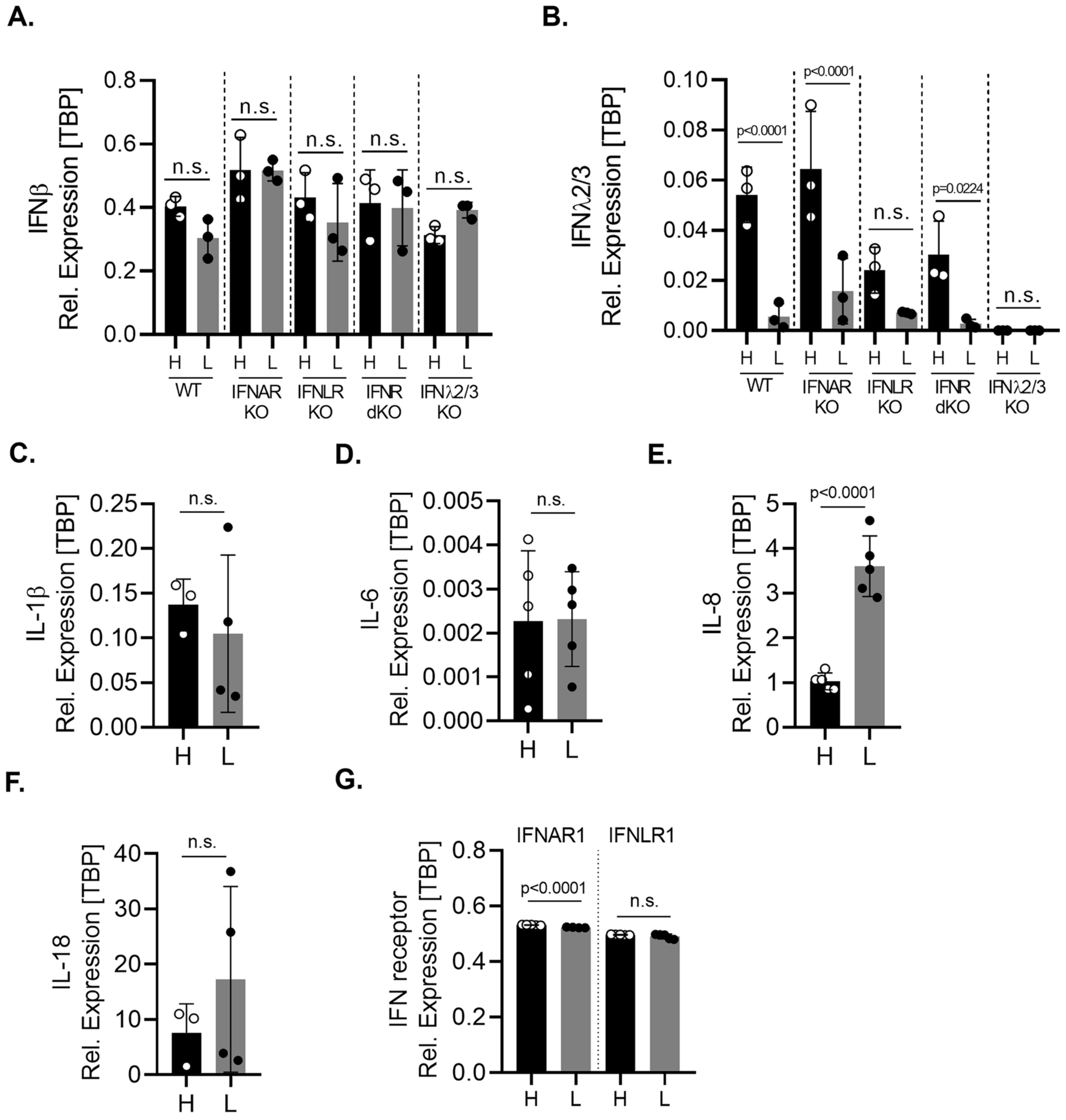

Figure EV2. Basal expression of type I IFNs and anti- and pro-inflammatory cytokines are not dependent on cell confluency.

T84 WT and T84 knock-out (KO) cells were seeded at high and low cellular density and the expression of the (**A**) IFNβ and (**B**) IFN-λ2/3 was addressed using qRT-PCR. T84 WT cells were seeded at high and low cellular densities and the relative expression of the cytokines IFNβ, IL-1β, IL-6, IL-18 and IL-8 was addressed using qRT-PCR. (**C**) IL-1β, (**D**) IL-6, (**E**) IL-8 and (**F**) IL-18. (**G**) The expression level of the type I IFN and type III IFN receptors IFNAR and IFNLR was assessed by qRT-PCR analysis of T84 cells grown at high and low cellular density. Relative expression normalized to TBP. $n \geq 3$ biological replicates. Statistical analysis was performed using unpaired *t* test between high and low density. n.s. indicates non-significant results ($P > 0.05$). Exact *P* values are shown on the plots when significant; otherwise, results are not significant. Error bars represent standard deviation with the mean as the center. Source data are available online for this figure.

 © The Author(s)

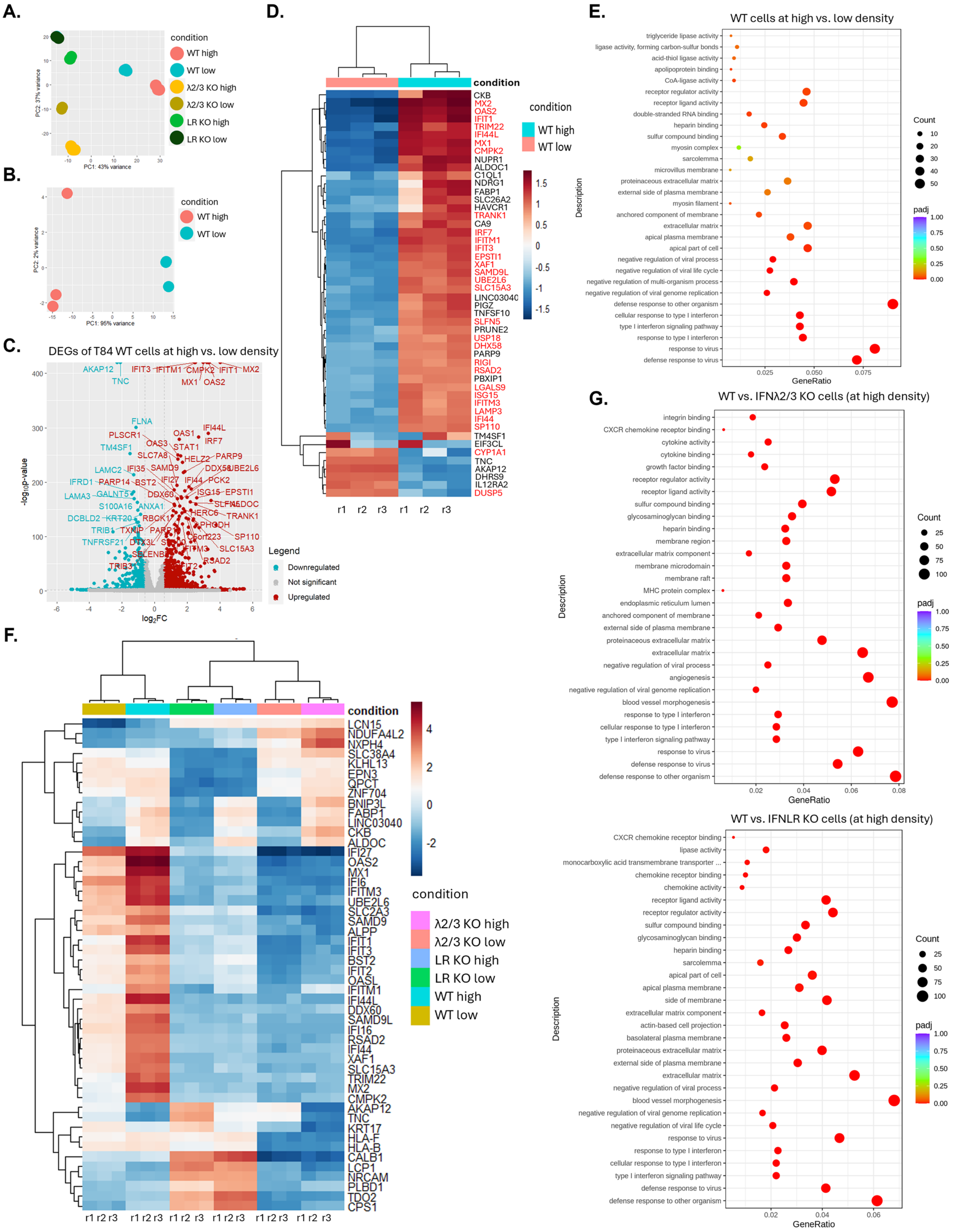

A.
condition
WT high
WT low
λ2/3 KO high
λ2/3 KO low
LR KO high
LR KO low
PC1: 43% variance
PC2: 37% variance
B.
condition
WT high
WT low
PC1: 95% variance
PC2: 2% variance
C.
DEGs of T84 WT cells at high vs. low density
Legend
Downregulated
Not significant
Upregulated
log2FC
-log10p-value
D.
condition
WT high
WT low
r1 r2 r3 r1 r2 r3
CKB
MX2
OAS2
IFIT1
TRIM22
IFI44L
MX1
CMPK2
NUPR1
ALDOC1
C1QL1
NDRG1
FABP1
SLC26A2
HAVCR1
TRANK1
CA9
IRF7
IFITM1
IFIT3
EPSTI1
XAF1
SAMD9L
UBE2L6
SLC15A3
LINC03040
PIGZ
TNFSF10
SLFN5
PRUNE2
USP18
DHX58
PARP9
RIGI
RSAD2
PBXIP1
LGALS9
ISG15
IFITM3
LAMP3
IFI44
SP110
TM4SF1
EIF3CL
CYP1A1
TNC
AKAP12
DHRS9
IL12RA2
DUSP5
E.
WT cells at high vs. low density
Description
GeneRatio
Count
padj
G.
WT vs. IFNλ2/3 KO cells (at high density)
WT vs. IFNLR KO cells (at high density)
Description
GeneRatio
Count
padj
F.
condition
λ2/3 KO high
λ2/3 KO low
LR KO high
LR KO low
WT high
WT low
r1 r2 r3 r1 r2 r3 r1 r2 r3 r1 r2 r3 r1 r2 r3 r1 r2 r3

Figure EV3. RNA-sequencing revealing the role of basal IFN-λ2/3 signaling is beyond its antiviral function.

T84 WT, IFN-λ2/3 KO and IFNLR KO cells were seeded at high and low density and subjected to RNA sequencing. (**A**, **B**) The PCA plot displays the distribution of (**A**) T84 WT, IFN-λ2/3 KO and IFNLR KO cells at high and low cell density and (**B**) only T84 WT cells at high and low density based on their gene expression profiles. Each point represents an individual sample, colored according to the experimental group. (**C**) The volcano plot illustrates the differential expression analysis results between T84 WT cells at high and low cell density. Each point represents a gene, plotted by its fold-change (*x* axis) and statistical significance (−log10 *P* value, *y* axis). Genes with significant differential expression ($P < 0.05$) are highlighted in red (upregulated) and blue (downregulated). Key genes of interest are labeled. (**D**) Heatmap displaying the expression levels of the top 50 differentially expressed genes across T84 WT cells at high and low density. ISGs were highlighted in red. (**E**) The enrichment of GO terms associated with the differentially expressed genes between T84 WT cells at high and low density. (**F**) **H**eatmap displaying the expression levels of the top 50 differentially expressed genes across T84 WT, IFN-λ2/3 KO and IFNLR KO cells at high and low density. (**G**) The enrichment of GO terms associated with the differentially expressed genes between (upper panel) T84 WT and IFN-λ2/3 and (lower panel) T84 WT and IFNLR KO cells at high density. (**D**, **F**) Rows represent genes, and columns represent samples, with hierarchical clustering applied to both dimensions. The color scale indicates relative expression levels, with red representing high expression and blue representing low expression. Clusters of co-expressed genes and samples are clearly visible, indicating distinct transcriptional signatures associated with the treatment. (**E**, **G**) GSEA plots were generated using a Kolmogorov–Smirnov-like statistic, as previously described (Subramanian et al, 2005).

© The Author(s)

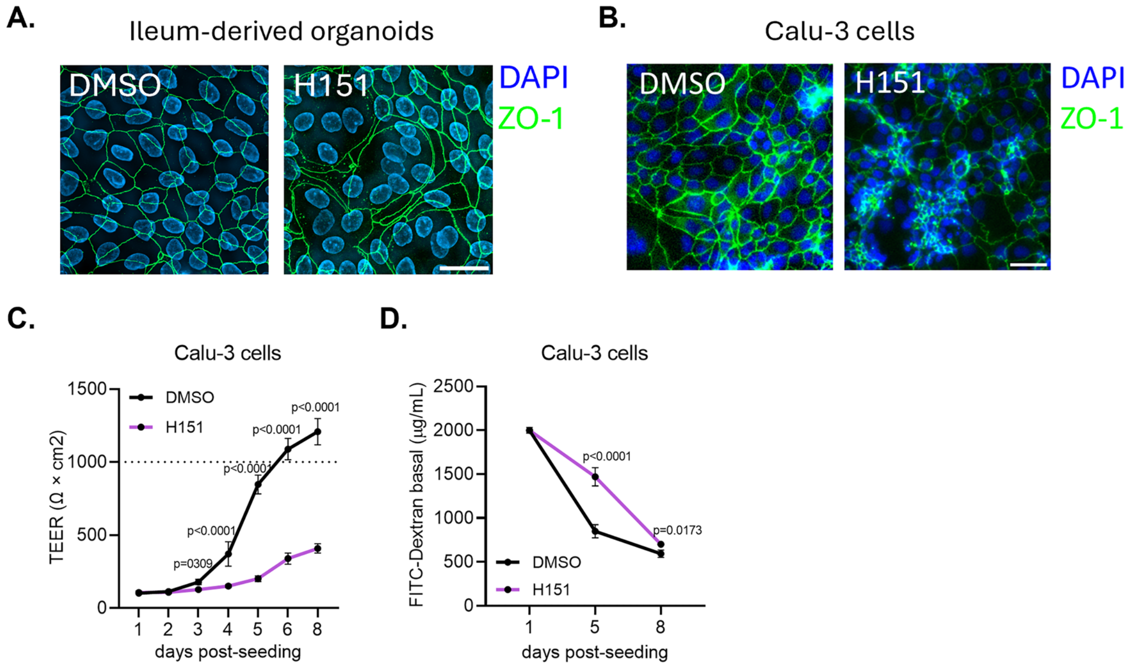

Figure EV4. Inhibition of STING impairs barrier formation in human primary intestinal epithelial cells and in human airway epithelial cells.

(**A**) Human ileum-derived organoids were mock-treated or treated with the STING inhibitor H151. Tight junctions were immunostained using an anti-ZO-1 antibody (green). Nuclei were stained using DAPI (blue). Scale bar = 25 µm. (**B**) Same as (A) but in the airway epithelial cells Calu-3 cells. Scale bar = 50 µm. (**C**) TEER measurement and (**D**) FITC-Dextran permeability assay for Calu-3 cells mock-treated or treated with the STING inhibitor H151. (**C**) Values > 1000 Ω/cm² (dotted line) shows that cells established barrier function. $n \geq 3$ biological replicates. Statistical analysis was performed using two-way ANOVA. n.s. indicates non-significant results ($P > 0.05$). Exact P values are shown on the plots when significant; otherwise, results are not significant. Error bars represent standard deviation with the mean as the center. Source data are available online for this figure.

© The Author(s)
